# Supplementary material for: Adaptation of the Client Diagnostic Questionnaire for East Africa
Source: PLOS Glob Public Health. 2024 Mar 19;4(3):e0001756. doi: 10.1371/journal.pgph.0001756 (PMC10950255; doi:10.1371/journal.pgph.0001756)
Supplement: S1 Questionnaire — (DOCX) [file pgph.0001756.s001.docx]

| **Syndemics**  **Client Diagnostic Questionnaire**  ***Instructions to interviewer:***  This questionnaire is designed to facilitate the recognition of the most common mental health problems found in HIV/AIDS primary care or other service settings: mood, anxiety, alcohol and drug abuse, PTSD and thought disorder.   1. Read questions as written. Additional probes may be used to ensure client understanding of the question or explore ambiguous answers. 2. For anything other than a “yes/no” answer, read the answer categories. The interviewer may need to assist the client in answering within the categories given. Never choose an answer category based on what you think the client means by their spoken response. 3. Be sure that the client is reporting symptoms experienced within the specified time period: past 4 weeks, past 6 months, or in some instances, past 30 days. 4. Within each module, proceed sequentially from question to question unless instructed either to skip to another question or to go to the next page. 5. At the end of each diagnostic module is a shaded area with instructions for scoring Positive Screen for each disorder. Scoring can be done by the interviewer or left for office use only. 6. A Summary Sheet is provided to record “positive screen” or “positive for syndrome” in the spaces provided for each diagnostic module. If no positive screen in any module, indicate in the space provided on the top of the summary sheet. 7. Space is also provided for interviewer observations and comments. Interviewer should write as detailed as possible description of positive answers to questions especially on psychosis screen. Where known, additional information that may account for symptoms (e.g. medical condition) or history of prior episodes or treatment should be indicated. 8. ***If Client indicates current suicidal feelings or becomes emotionally upset or agitated during interview, please contact a study supervisor immediately.*** |
| --- |
|  |
| ***Kumtambulisha mteja*** |
| Haya maswali yatatusaidia kuelewa zaidi shida ambazo huenda ukawa nazo. Tunauliza haya maswali kwa kila mmoja ili tupate picha bora ya usaidizi au msaada ambao tunaweza kukupatia. Tafadhali jaribu kujibu kila swali. Majibu yako yote yatakuwa ya siri kikamilifu. |
| 1.    ***Maelezo ya jumla:*** Unapofikiria karibu miezi sita iliyopita, yaani kutoka mwezi wa ……..(rejea tarehe, miezi 6 kabla ya mahojiano), mambo yamekuwa yakikuendeaje kihisia? Kuna muda ambao ulikuwa na huzuni sana au kusononeka? Na je, upo wakati ulikuwa na hofu sana, mwenye kuogopa, au kuwa na wasiwasi kuhusu mambo? Kuna wakati ambao ulikuwa mchangamfu sana au kusisimka kiasi kwamba haungeliweza kupunguza mwendo? |
| 2.    Kuna jambo lolote lililokufanyikia kwa wakati huo ambalo lilihusiana na hisia yako (au kutenda) kwa njia hii (huzuni, wasiwasi, kusisimka na kadhalika…rejelea dalili)? Kuna jambo lolote hasa lilikuwa gumu au la kutatiza kwako? |
| 3. Kwa miezi sita iliyopita, ulizungumza na mtu yeyote kuhusu shida zako za kihisia, hofu au jinsi ulivyokuwa ujahisi au kutenda? Kama NDIVYO, ni nani uliyezungumza naye? *(Hoji zaidi)* Je, ulizungumza na mtaalamu kama daktari au mshauri? Walisemaje kulihusu jambo hilo? |
| ***Anayehoji:*** *Iwapo mteja anaeleza dalili au historia ya matibabu, mjulishe ya kwamba utazungumzia kuhusu jambo hili kwa kina baadaye katika mahojiano. Lazima maswali yote ya uchunguzi na ya dalili yaulizwe hata kama yalikuwa yamezungumziwa katika maelezo ya jumla. Thibitisha majibu yanayojulikana.* |
|  |
| **Sasa nitakuukiza maswali kuhusu hisia zako. Katika mwezi uliopita (wiki 4 zilizopita) kuna wakati ambapo...**  **La, Siku Zaidi Karibu**  **Hata Nyingi Ya kila Siku**  **Kamwe** |
|  |
| ***1.****Ulikuwa unahisi huzuni, kusononeka au kukosa matumaini?* 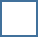 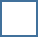 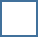 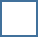 *KAMA NDIVYO, ni mara ngapi ulihisi hivyo?*? |
| ***2****. Hukuwa na hamu au raha ya kufanya mambo?* 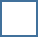 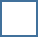 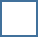 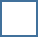  *KAMA NDIVYO, ni mara ngapi ulihisi hivyo?* |
| **Iwapo majibu ya mteja ni "La, haijatokea kabisa" kwa maswali yote, mawili, endelea kwa ukurasa unaofuata** |
| 3. Ni lini ulianza kuhisi hivi (kipindi cha hivi karibuni)? |
| 4. Hali hii ilidumu kwa muda gani__________kwa wiki mbili hivi? **Ndiyo** **LA** |
| **Katika kipindi hicho, ni mara ngapi ulikuwa (umekuwa) ukisumbuliwa na:**    **La, Siku Zaidi Karibu**  **Hata Nyingi ya nusu Kila siku**  **Kamwe Masiku** |
| 5. Matatizo ya kupata usingizi au kuweza kulala? Au kulala sana? |
| 6. Kujihisi mchovu au kuwa mdhaifu |
| 7. Kutokuwa na hamu ya kula? Au kukula sana? |
| 8. Kuhisi vibaya kujihusu au kujiona kuwa umeshindwa kabisa  au umejiangusha au kuiangusha familia yako |
| **9**. Shida kuwa makini kwa mambo kama vile kusoma gazeti,  kutazama runinga, kusikiliza mtu akikupa mwelekezo,  au kufuatilia maongeo |
| 10. Kutembea au kuongea polepole kiasi kwamba watu wengine  wangetambua? Au kinyume- kutotulia kiasi kwamba  ulikuwa unatembe tembea zaidi ya kawaida? |
| 11.  Ulikuwa na mawazo ya kwamba heri ungekuwa umekufa    au mawazo ya kujiumiza mwenyewe kwa njia fulani?  Maj Dep Syn if 2 weeks (Q4) is “yes” (AND) answer to question 1 or 2 is shaded (AND) 5+ of answers to any of Q. 1, 2, 5 - 11 are shaded; Other Dep Syn same but only 2+ of the answers to Q. 1, 2, 5 - 11 are shaded. |
|  |
| **Sasa nitakuukiza maswali kuhusu wasiwasi......**  **NDIO LA** |
| 1. Katika wiki 4 zilizopita, umekuwa na shinikizo la wasiwasi-   kuhisi uoga au kushtuka kwa ghafla? |
| **Kama mteja atajibu "LA" nenda kwa ukurasa unaofuata** |
| 1. Je, hili limewahi kufanyika awali? |
| 3. Je, baadhi ya shinikizo hizi huja ghafla-  yaani katika hali ambayo hutaraji kuwa na wasiwasi au kukosa starehe? |
| 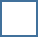  4. Je, shinikizo hizi hukutatiza sana? Una hofia kuwa na shinikizo jingine? |
| **Fikiria kuhusu shinikizo la mwisho lililokuwa baya zaidi** |
| 5. Ulipungukiwa na pumzi? |
| 6. Moyo wako ulidunda, ulipiga kwa kishindo au kuruka mpigo. |
| 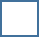7. Ulikuwa na uchungu au shinikizo kifuani? |
| 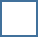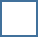8. Ulitokwa jasho? |
| 9. Ulihisi kusakamwa (kitu kukwama kooni?)? |
| 10. Ulihisi joto au kibaridi kwa ghafla?? |
| 11. Ulikuwa na kichefuchefu au kuhisi msokoto wa tumbo  au kuhisi kana kwamba utaendesha?? |
| 12. Ulihisi kizunguzungu, kutokuwa thabiti, au kuzimia?? |
| 13. Je, ulihisi kufa ganzi (au kuhisi kama kudungwadungwa kwa sindano)  katika sehemu za mwili wako?? |
| 14. Ulitetemeka? |
| 15. Ulikuwa na uoga kuwa utaaga?  Pan Syn if answers to Q. 1,2,3 and 4 are ‘Yes’ (AND) 4+ symptoms during an attack (Q. 5-15) |
|  |
| **Katika wiki 4 zilizopita, ni mara ngapi umesumbuliwa na:**  **La, Siku Zaidi Karibu**  **Hata Nyingi Ya Kila Siku**  **Kamwe** |
| 1. Kuhisi kubabaika, hofu au kuwa na wasiwasi kuhusu   mambo kadha wa kadha? |
| Kama mteja atajibu "LA" nenda kwa ukurasa unaofuata. |
| 2. Kuhisi kutotulia kiasi kwamba huwezi kushinda mahali pamoja? |
| 3. Kuchoka kwa urahisi sana? |
| 4. Mvuto, uchungu au maumivu kwa misuli |
| 5. Shida kupata usingizi au kubakia usingizini? |
| 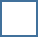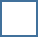6.  Shida kuwa makini kwa mambo kama vile kusoma gazeti,  kutazama runinga, kusikiliza mtu akikupa mwelekezo,  au kufuatilia maongeo |
| 7. Kukasirika au kughadhabika kwa urahisi? |
| **Other Anx Syn if answer to Q. 1 is shaded (AND) 3+ answers to Q. 2-7 are shaded** |
| **Yafuatayo ni maswali kuhusu unywaji wa pombe na matumizi ya madawa mengine ya kulevya. Tunauliza maswali haya kama maelezo ya hali ya afya ya kila mtu. Yote unayoniambia yatahifadhiwa kwa siri**. |
| 1. Katika miezi sita iliyopita, ni mara ngapi umekuwa ukinywa bia, divai au mvinyo?   Hakuna Chini ya Mara Moja Kila Mwezi Kila Wiki Mara Tatu Kila Siku  Kwa Mwezi Kwa Mwezi |
| **Kama mteja hanywi pombe, nenda kwa swali la mwisho la pombe -swali la 13 ukurasa unaofuata.** |
| *****Tafadhali tumia maagizo yaliyoko kwa kijikaratasi "Hesabu ya vinywaji vilivyonywiwa" katika swali la 2***** |
| 1. Ni vinywaji vingapi unavyotumia kwa zile siku unazokunywa?   Moja Mbili Tatu Nne Tano Zaidi ya Tano |
| Je, jambo lolote katika haya yafuatayo limekufanyikia zaidi ya mara moja katika miezi 6 iliyopita, yaani kutoka (…………………...) hadi leo? (Jaza tarehe miezi 6 kabla ya mahojiano.  **NDIO LA** |
| 1. Ulikunywa pombe ingawa daktari alipendekeza uache kunywa   kwa sababu ya shida na afya yako? |
| 1. Ulikunywa pombe, ulikuwa umelewa pombe au kuchoka kutokana na   kunywa pombe, ulipokuwa kazini, shuleni au ukichunga watoto  au katika majukumu mengine? |
| 1. Ulikosa, au kuchelewa kwa shughuli muhimu kwa sababu ulikuwa   ukinywa pombe au ulikuwa mchovu kutokana na kunywa pombe? |
| 6. Ulikuwa na shida ya kuelewana na watu wengine ulipokuwa ukinywa? |
| 1. Uliendesha gari (gari dogo, lori, pikipiki) au ulifanya kazi na mashine nzito   baada ya kunywa vinywaji kadhaa au baada ya kunywa sana? |
| Alc Abu if 1+ answers to Q. 3-7 are Yes (OR) 5+ drinks a day weekly or more often |
| **Kwa siku 30 zilizopita, yaani tangu wakati huu mwezi wa (………….) (mwezi kabla ya mahojiano)** |
| 1. Ni siku ngapi ulikunywa aina yoyote ya pombe? |
| **Kama mteja hakunywa pombe katika siku 30 zilizopita, nenda kwa swali la mwisho la pombe -swali la 13 hapo chini** |
|  |
| **Katika siku 30 zilizopita…**  **NDIO LA** |
| 1. Umefikiria upunguze unywaji wako wa pombe? |
| 10. Mtu yeyote amelalamika kuhusu unywaji wako wa pombe? |
| 1. Umewahi jihisi mwenye hatia au kutofurahia unywaji wako wa pombe? |
| 1. Ipo siku yoyote ambamo ulikunywa vinywaji tano au zaidi; bia, divai   au mvinyo?? |
| **ULIZA KILA MTU** |
| 1. 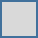Je, wewe au mtu yeyote aliye na uhusiano wa karibu nawe amewahi   fikiria ya kwamba una shida na unywaji wa pombe? |
| Alc Abu 30 day if 2+ answers to questions 9-12 are YES |

| **Hapa kuna maswali chache kuhusu utumizi wa madawa. (Kumbusha mteja kuhusu usiri). Kumbuka ya kwamba yote utakayoniambia ni siri na yatalindwa (hayataelezwa mtu yeyote aziyehusika). Umewahitumia madawa yoyote kati ya yafuatayo, hata mara moja.** |
| --- |
|  |
| \|  \| **Umewahi**  **Tumia** \| \| **Iwapo Ndiyo kwa dawa yoyote uliza:**  **Kwa Miezi 6 iliyopita, ni mara ngapi ulitumia (Dawa)?** \| \| \| \| \| \| \| --- \| --- \| --- \| --- \| --- \| --- \| --- \| --- \| --- \| \| **Ndio** \| **la** \| **Sijawahi** \| **Chini ya mara moja kwa mwezi** \| **Kila mwezi** \| **Kila wiki** \| **Mara 3 kwa wiki** \| **Kila siku** \| \| 1. Marijuana (hashish, cannabis, weed, bhang, bangi, ganja, sensi, boza, ikhendi)) \|  \|  \|  \|  \|  \|  \|  \|  \| \| 1. Kokain \|  \|  \|  \|  \|  \|  \|  \|  \| \| 1. Crack, freebase \|  \|  \|  \|  \|  \|  \|  \|  \| \| 1. Heroin, brown, sugar, white cap, white crest, unga \|  \|  \|  \|  \|  \|  \|  \|  \| \| 1. Methadone bila ya kuandikiwa au zaidi ya vile daktari alikueleza. \|  \|  \|  \|  \|  \|  \|  \|  \| \| 1. Sedatives (Vitulizi) or downers (Valium, stilnox, cough syrup) bila ya kuandikiwa au zaidi ya vile daktari alikueleza \|  \|  \|  \|  \|  \|  \|  \|  \| \| 1. Stimulants (Visisimuzi) (methamphetamine, uppers, speed, barafu) bila kuandikiwa au zaidi ya vile daktari alikueleza… \|  \|  \|  \|  \|  \|  \|  \|  \| \| 1. Hallucinogens (PCP, angeldust, ecstacy, uyoga, LSD) …. dawa yenyeminafanya mtu kuona, kusikia na kuona vitu ambavyo haviko au sio vya ukweli.) \|  \|  \|  \|  \|  \|  \|  \|  \| \| 1. Kunusa au Kuvuta chochote ili ulewe (dawa ya kupuliza, glue) …. \|  \|  \|  \|  \|  \|  \|  \|  \| \| 1. Mengine (angalia Kiambatisho 2) \|  \|  \|  \|  \|  \|  \|  \|  \| |

|  |
| --- |
|  |
| *IWAPO AMAWAHITUMIA MADAWA YEYOTE.:* **NDIO LA** |
| 1. Umewahi kujidunga au kudungiwa dawa au kuwekewa chini   ya ngozi kupitia sindano hata mara moja? |
| *IWAPO AMEWAHITUMIA SINDANO:* |
| 1. Umewahi kujidunga au kudungiwa dawa au kuwekewa chini ya ngozi   kupitia sindano wakati wowote kwa miezi sita iliyopita?  Iwapo ni ndio, ni dawa gani? |
| **Iwapo Hakuna Utumizi wa Dawa Kwa Miezi 6 nenda kurasa 11 TRAUMA.** |
|  |
| **Uliza wateja wote ambao wametumia dawa yoyote kwa miezi 6 iliyopita.** |
| Kuna lolote kati ya mambo yafuatayo limekufanyikia zaidi ya mara moja  kwa miezi 6 iliyopita, ambayo ni kuanzia (____________) hadi leo??  **NDIO LA** |
| 1. Ulitumia madawa hata kama daktari alikupendekezea uache kutumia kwa   sababu ya shida na afya yako? |
| 1. Ulitumia madawa, ulilewa au kuwa na uchovu wa ulevi wa madawa   ulipokuwa kazini, shuleni au kuwalea watoto ama majukumu yoyote. |
| 1. Ulikosa au kuchelewa kwa shuguli muhimu kwa sababu ulikuwa   unatumia madawa au uchovu kutokana na madawa ya kulevya? |
| 1. Ulikuwa na shida kuelewana na watu wengine ulipokuwa unatumia madawa? |
|  |
| 17. Uliendesha gari baada ya kutumia madawa? |
| 1. Ulikuwa na shida za kisheria kwa sababu ya utumizi wa madawa   (mkwaruzano na polisi, kupelekwa kortini, kushtakiwa na mtu yeyote,  shida zozote na sheria) …. |
| DRUG ABU if 1+ answers to Q 12 - Q 18 are Yes (OR) Heroin, Coke/Crack or Methamphetamine 3+ per week |
| **KWA SIKU 30 ILIYOPITA, tangu siku hii kwa (________) (Mwezi kabla ya mahojiano).** |
| \| Ni siku ngapi ulitumia…...… \| \| --- \| \|  \| |
| 1. Marijuana |
| 1. Kokaine |
| 1. Crack |
| 1. Heroine au speedball |
| 1. Sedatives, (Vitulizi) downers |
| 1. Stimulants, (Visisimuzi) uppers |
| 1. Hallucinogens   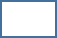(dawa yenye inafanya mtu kuona,  kusikia na kuhisi vitu ambavy  haviko au sio vya ukweli) |
| 21. Inhalants (dawa ya kupuliza) |
| **Iwapo mteja hakuwahi tumia dawa siku 30 zilizopita nenda kurasa ifuatayo.** |
| **Kwa siku 30 zilizopita**….  **NDIO LA** |
| 1. Umewahi kufikiria kupunguza utumizi wako wa madawa? |
| 1. Mtu yeyote amewahi lalamika kuhusu utumizi wako wa madawa? |
| 1. Umewahi hisi kujuta au kukasirika kuhusu utumizi wako wa madawa? |
| 25. Umetumia dawa yoyote mara 3 au zaidi kwa wiki au kila mara?? |
|  |
| ULIZA KILA MTU |
| **Sasa hapa kuna maswali kuhusu mambo ya kutisha au ya kuogofya ambayo huenda yamekufanyikia.** |
| Watu mara nyingi hukumbana na mambo ya kutisha. Namaanisha matukio ya kutisha na ya kuogofya. Ninaenda kusoma orodha ya matukio ambayo wakati mwingine yanaweza fanyikia watu. Tafadhali niambie iwapo umewahi kumbana nayo.... |
| **NDIO LA**   1. Ajali mbaya (nyumbani, gari, pikipiki au moto nyumbani au kazini. . .. |
| 1. Janga kama vile kimbunga, tetemeko la ardhi, dhoruba kali, mafuriko   au majanga sawiya.… |
| 1. Kupambana moja kwa moja katika vita |
| 1. Kushambuliwa kimwili au kudhulumiwa katika maisha yako ya utu uzima   na mpenziyo. |
| 1. Kushambuliwa kimwili, kudhulumiwa, kuibiwa kimabavu katika maiasha yako   ya utu uzima na mtu kando na mpenziyo. |
| 1. Kushambuliwa kimwili au kudhulumiwa ukiwa mtoto…… |
| 1. 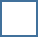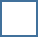Kushuhudia watu wakipigana au kuumizana katika familia yako ulipokuwa unakua… |
| 1. Kushambuliwa kingono au kunajiziwa ukiwa mtu mzima. . . |
| 1. Kushambuliwa kingono au kunajisiwa ukiwa mtoto. . .. |
| 1. Kuona mtu akishambuliwa kimwili au kudhulumiwa. |
| 1. Kuona mtu akijeruhiwa vibaya au kuuwawa kinyama. . .. |
| 1. Kupoteza mtoto kupitia kifo. . .. |
| 1. Kupoteza mimba. |
| 1. Jambo jingine la kutisha au kuogofya ambalolimeshawahi kutendekea.   Bainisha……. |
| **Iwapo mteja atajibu "LA" kwa maswali yote nenda kwa kurasa la 13, PSY** |
| **Iwapo mteja atajibu "NDIO" kwa swali moja au zaidi nenda kwa KURASA INAYOFUATA** |
|  |
| **Iwapo mteja atajibu "NDIO" kwa tukio MOJA TU iliyoorodheshwa kwa kurasa ya awali, Uliza swali la 1A.** |
| 1A. Umeniambia kuhusu wakati_____________ (taja tukio). Ningependa kukuuliza zaidi kuhusu tukio hili…………...**ruka kwa swali la 2.** |
| **Iwapo mteja atajibu "NDIO" kwa ZAIDI YA TUKIO MOJA kwa kurasa ya hapo awali, Uliza swali 1B.** |
| 1B. Umeniambia kuhusu baadhi ya matukio ambayo yamekufanyikia. Ni gani kati ya matukio haya yalikuwa ya kutisha zaidi au ya kuogofya kwako? (Bainisha tukio au mfululizo wa matukio ambayo mteja anataja) |
| **Ningependa kukuuliza zaidi kuhusu tukio hili (mfululizo wa matukio) …...** |
| 1. Uliogopa kiasi gani?   Hata Kamwe Kidogo tu. Vibaya Vibaya sana Kushtuka Kiwango cha Kufa |
| **Kwa miezi sita iliyopita**  **NDIO LA** |
| 1. Unaendelea kukumbuka tukio hilo hata kama hautaki? |
| 1. Unakuwa na ndoto mbaya kulihusu tukio hilo? |
| 5. Je, mambo yanayokukumbusha kulihusu tukio hilo yanakufanya kutofurahi  au kusumbuka? |
| 6. Huwa unakuwa na kumbukumbu - hisia ya ghafla kuwa tukio lilikuwa  linafanyika tena? |
| 7. Je, una wasiwasi sana kuwa linaweza kufanyika tena? |
| 8. Je, unajiepusha na vitu ambavyo vinakukumbusha kulihusu? |
| 9. Je, wakati mwingine unakuwa na shida kukumbuka ni nini haswa ilifanyika? |
| 1. Je, una hisi kuwa pekee hata wakati unapokuwa na watu wengine au   kuhisi kutengwa na watu?? |
| 11. Je, una hisi kufa ganzi au kana kwamba hauna hisia nzito tena kwa chochote?? |
| 12. Je, unakosa utulivu au kuwa na wasiwasi au kushtushwa kwa urahisi au  kuwa mwangalifu kila mara pasipo sababu?? |
| ******Iwapo mteja atajibu "NDIO" kwa tukio ZAIDI YA MOJA kwa kurasa ya awali, uliza swali 1C kwa Kiambatanisho cha 3 ****** |
| PTS Syn if answer to 2 is “Bad” or worse (AND) 1+ answers to Q 3-6 (AND) 2+ answers to Q.8-11 are YES |
| **Sasa nitakuuliza kuhusu baadhi ya imani na hisia ambazo watu huwa nazo. Baadhi ya watu wana hizi hisia na imani baada ya kunywa pombe au kutumia madawa. Ningependa kujua kama umewahi kuwa na baadhi ya hizi imani au hisia kwa WIKI 4 ZILIZOPITA (siku 30 zilizopita) wakati ambapo HAUJAKUNYWA pombe au kutumia madawa.** |
| **Kwa wiki 4 zilizopita, ni mara ngapi……...**  **Zaidi**  **Mara ya Mara**  **Hakuna Moja Moja** |
|  |
| 1. Umewahi kusikia kelele au sauti ambazo watu wengine husema  hawaziskii? Kama NDIO: Niambie ilikuwa nini ulichosikia?  Kama ni sauti: hiyo sauti ilisema nini? Ilikuambia hufanye nini?  Ni kama hiyo sauti iko ndani ya kichwa chakoau ilitokea nje?? |
| 2. Umewahi hisi kwamba kulikuwa na watu walitaka kukudhuru  au kukuumiza? Kama NDIO: Hawa watu ni akina nani?  Ni kwa nini wanataka kukuumiza? Je? Wasiwasi  au uoga wako kuhusu jambo hili hukufanya uone/upate ugumu  kutoka nyumbani kwako au mahali unapolala. |
|  |
| 3. Umewahi hisi kuna jambo lisilokuwa la kawaida au lisilo la  kawaida linaloendelea katika mazingira yako au  mambo yanayokuzingira yanabadilika. Kama NDIO:  unaweza kunieleza lolote kulihusu? Je, Unaona  kama watu wanakupangia njama? Je, mambo yanakaa  kuwa na maana maalum kwako? Kama vile nambari  au saini/vielekazo za barabarani au vitu kama hivyo? |
| 4. Je, umekuwa na maono au kuona vitu ambavyo watu wengine  wanasema hawavioni? Kama NDIO: Niambie kuhusu ulichoona.  Je, hili linafanyika ukiwa macho? Je hufanyika wapi?  Je, unaona mtu ambaye alikufa hivi karibuni? |
| 5. Je, umehisi kuwa una uwezo maalum/ wakipekee ambao  watu wengine hawana? Kama NDIO: Nieleze kuhusu uwezo huo.  Uwezo huo una tofauti gani na ule wa watu wengine?  Je, umetumia vipi huo uwezo? |
| 6. Je, Umewahi kufikiria kuwa umeingiliwa na pepo ama/au shetani?  Kama NDIO: Je, unaweza kunieleza kuhusu jambo hilo? Je, pepo/shetani  alikufanya ufanye jambo lolote? Jambo lipi? |
|  |
| **Kwa wiki 4 zilizopita, ni mara ngapi……...**  **Zaidi**  **Mara ya Mara**  **Hakuna Moja Moja** |
| 7. Je, umewahi hisi kuwa mawazo yako yamechukuliwa kutoka  kwako na kitu/mtu mwingine? Kama NDIO: Ni nani au ni nini  huchukuwa mawazo yako? Je, unafikiria hili hufanyikaje? |
| 8. Je, Umewahi kuwa na mawazo au mafikira ambayo hakuna  mtu mwingine yeyote angeweza kuelewa. Kama NDIO: Nieleze  kuhusu haya mawazo. Je, unajuaje ya kwamba hakuna mtu mwingine  yeyote angeweza kuelewa? |
| 9. Je, Umewahi hisi kuwa mawazo yasiyo yako yamewekwa  katika kichwa chako? Kama NDIO: Baadhi ya mawazo haya ni yapi?  Je, unafikiria yanaingiaje kwa kichwa chako? |
| 10. Je, Umewahi hisi kuwa akili yako ilichukuliwa na nguvu  ambazo haungeweza kuzuia? Kama NDIO: Ni nani au nini huchukua  maamlaka juu ya akili yako? Unafikiri hili linafanyikaje? |
| **Maoni au Uchunguzi wa ziada…...** |
|  |
| Psy Screen Positive if 2+ answers are shaded (OR) 3+ symptoms one time only. Do not score unless experiences described are implausible and outside of ordinary or culturally supported experiences  **Maswali yafuatayo ni kuhusu huduma tofauti ambazo huenda umepokea (Thibitisha habari iwapo inajulikana)** |
| 1. Je, Umewahi kuongea na mtaalamu wa afya ya akili kama vile daktari wa akili, mwanasaikologia, ama mfanya kazi wa jamii aliye na mafunzo maalum (aliye na shahada), kuhusu shida za kihisia, kuwa na wasiwasi au jinsi unavyojihisi au tabia zako.?   **NDIO LA Kama NDIO_________** Mtaalamu wa afya ya akili alisema nini?  Uliza kama mhojiwa anajua ugonjwa wake? |
| 1. Umewahi kuongea na mtu ambaye hakutajwa katika swali lililotangulia kuhusu shida za kihisia, wasiwasi wako au jinsi ambavyo ulikuwa unahisi au tabia zako?   **NDIO LA Kama NDIO _________** (Mtu huyo) Alisema nini? |
| 1. Umewahi kuandikiwa dawa ya kukusaidia kwa shida za kihisia au kisaikolojia au jinsi ulivyokuwa unahisi au tabia zako?   **NDIO LA Kama NDIO:** Ni dawa gani? |
| 1. Umewahi kulazwa hospitalini kwa sababu ya shida za kihisia au kisaikolojia au jinsi ulivyokuwa unahisi au tabia zako?   **NDIO LA Kama NDIO:** Ilikuwa lini? Ni kwa nini ulilazwa? |
| 1. Umewahi pata matibabu yeyote ya shida ya pombe au dawa za kulevya?   **NDIO LA Kama NDIO**: Ilikuwa lini? Ulipata matibabu ya aina gani? |
| 1. Kwa muda wa miezi sita iliyopita, umewahi pokea usaidizi wowote kuhusu changamoto za kihisia au kisaikolojia kama vile kuongea na mtaalamu wa kisaikolojia au daktari wa akili au kutumia tembe au kulazwa hospitalini   ***Chora duara kwa yote yanayofaa*** |
| a. Ulipokea matibabu ya kutolazwa au ushauri kwa shida ya kisaikolojia ____________________ |
| b. Ulipokea matibabu kwa sababou ya pombe au dawa za kulevya________________________ |
| c. Madawa/miti shamba (taja) _____________________________________________________ |
| d. Kulazwa hospitalini? __________________________________________________________ |
| e. Mengine (taja) ______________________________________________________________ |
| 1. Je, kuna jambo lingine lolote la muhimu ungependa kunieleza lililofanyika kwa muda wa miezi sita iliyopita kuhusu hali, hisia, mawazo au tabia zako? |
|  |
| (Optional Demographic Questions) |
| **Hatimaye, kuna maswali machache kukuhusu.** |
| **1.   Siku yako ya kuzaliwa ni lini? ____/_____/______** Mwezi/siku/mwaka |
| **2. Jinsia ya mteja** (Thibitisha na mteja) |
| a.  Mume |
| b.  Mke |
| c.  Jinsia badala (transgender) |
| **3.   Ulizaliwa wapi ______________________ (**Kaunti/wilaya) |
| **4. Ni lugha gani unapendelea kuongea? (**Chagua moja**)** |
| a.  Kiingereza |
| b.  Swahili |
| c. Lugha za Kenya (taja) |
| d. Zingine (taja) |
| **5. Ulifika kiwango gani cha shule? Ni kiwango gani cha juu cha diploma au shahada ulichopata, Iwapo ipo lolote?** |
| a.  Hakuna |
| b.  Shule ya msingi (hukumaliza) |
| c.  Shule ya msingi (ulimaliza) |
| d.  Shule ya upili (hukumaliza) |
| d.  Shule ya Upili (ulimaliza)) |
| e. chuo Kikuu (taja shahada) |
| f. Mafunzo ya Kitaaluma (taja) |
| g. Shule ya kiufundi (taja) |
| h. Mengine (taja) |
| **6. Unajitambuaje?** |
| a. Wanaume wanaohusiana kimapenzi na wanaume wenzao |
| b. Wanawake wanaohusiana kimapenzi na wanawake wenzao |
| c. Jinsia zote, kuvutiwa na waume na wake |
| d. Anayevutiwa kimapenzi na mtu wa jinsia tofauti na yake |
| e. Sina uhakika/sijaamua/nabadili |
| f. Naafadhalisha kutosema |
| **7. Celi yako ya T ya hivi karibuni au hesabu ya CD4?** |
| *Iwapo mteja anataja nambari, andika hapa ndani \|___\|____\|_____\| au jingine tumia kodi hapa chin* |
| a. 0-100 |
| b. 101-200 |
| c. 201-300 |
| d. 301-500 |
| e. Zaidi ya 500 |
| f. Sijui hesabu ya celi ya T lakini niliambiwa ilikuwa "Nzuri" |
| g. Sijui hesabu ya celi ya T lakini niliambiwa ilikuwa "Mbaya" |
| h. Sijui hesabu ya celi ya T hata kidogo/Sikumbuki matokeo ya pimo |
| i. Mteja hajawahi kuwa na pimo ya celi ya T ya CD4 |

|  |
| --- |
| **KARATASI YA MUHTASARI (JAZA BAADA YA MAHOJIANO)** |
| Tathmini kila sehemu ya orodha ya maswali ya uchunguzi na peana alama ya CDQ kufuata maelezo kwa sanduku chini ya ukurasa mwishoni mwa sehemu yoyote ya utambuzi. Rekodi matatizo yote ambayo mteja anapata chanya katika karatasi hii. |
| Iwapo mteja hatapata chanya kwa kila sehemu weka alama ya kusahihisha hapa:  ❏ **Hakuna chanya kwa kila sehemu** |
| **MATATIZO YA HUZUNI** |
| ❏ Chanya kwa matatizo Makuu ya Huzuni |
| ❏ Chanya kwa matatizo Mengine ya Huzuni |
| Je dalili za mteja za huzuni ni kutokana kwa kifo cha mpenwa? Je, dalili zinawezakusababishwa na ugonjwa wa mwili, matibabu au utumizi wa madawa? Je mteja amewahi kupokea matibabu kwa matatizo? Maoni mengine: |
| **MATATIZO YA WASIWASI** |
| ❏ Chanya kwa dalili ya Hofu |
| ❏ Chanya kwa dalili ya Hofu ya ujumla |
| Je dalili inawezasababishwa na ugonjwa wa mwili, matibabu, au utumizi wa madawa? Je, mteja amewahipokea matibabu kwa matatizo? Maoni mengine: |
| **URAIBU WA POMBE** |
| ❏ Chanya kwa Uraibu wa Pombe kwa muda wa miezi 6 iliyopita.  ❏ Chanya kwa Uraibu wa Pombe kwa muda wa siku 30 iliyopita |
| Je, mteja amewahi kupokea matibabu kwa uraibu wa pombe/kutegemea pombe? Je, mteja amekuwa katika mazingira ya kuzuiliwa (Kama gereza, hospitali) wakati wowote kwa muda wa miezi 6 iliyopita? Kwa muda wa siku 30 iliyopita? Maoni mengine: |
|  |
| **URAIBU WA MADAWA** |
| ❏ Chanya kwa Uraibu wa Madawa kwa muda wa miezi 6 iliyopita- Orodhesha dawa/madawa uliotumia vibaya ______________________________ |
| ❏ Chanya kwa Uraibu wa Madawa kwa muda wa siku 30 zilizopita- Orodhesha dawa/madawa uliotumia vibaya: _______________________________  Je, mteja amewahi kupokea matibabu kwa uraibu wa madawa/kutegemea madawa? Je, mteja amekuwa katika mazingira ya kuzuiliwa (Kama gereza, hospitali) wakati wowote kwa muda wa miezi 6 iliyopita? Kwa muda wa siku 30 iliyopita? Maoni mengine: |
|  |
|  |
|  |
| **MATATIZO YA BAADA YA MKASA,** |
| ❏ Chanya kwa uchunguzi wa matatizo ya baada ya mkasa |
| Eleza matukio ya kutisha Je, dalili zinawezakusababishwa na ugonjwa wa wa mwili, matibabu au utumizi wa madawa? Je, mteja amewahi kupokea matibabu kwa matatizo? Maoni mengine: |
| **SAIKOSIA** |
| ❏ Chanya kwa uchunguzi wa saikosia |
| Eleza dalili. Je, dalili zimesababishwa na ugonjwa wa wa mwili, matibabu au utumizi wa madawa? Je, mteja amewahi kupokea matibabu kwa matatizo? Maoni mengine: |
| **TAJIRIBA YA MATIBABU** |
| ❏ Mteja amepata matibabu ya mtaalamu wa afya ya akili au ameandikiwa madawa ya ugonjwa wa akili kwa muda wa miezi 6 iliyopita. |
| ❏ Mteja anapokea kwa sasa matibabu ya mtaalamu wa afya ya kiakili au ameandikiwa madawa ya ugonjwa wa akili. Tarehe ya matibabu? Je, matibabu yamekamilika? Je, mteja anafuatilia/alifuatilia mpango wa matibabu? Maoni mengine: |
