# Supplementary material for: Adaptation of the Client Diagnostic Questionnaire for East Africa
Source: PLOS Glob Public Health. 2024 Mar 19;4(3):e0001756. doi: 10.1371/journal.pgph.0001756 (PMC10950255; doi:10.1371/journal.pgph.0001756)
Supplement: S2 Questionnaire — (DOC) [file pgph.0001756.s002.doc]

**ETAC/ CDQ- OBOKE MACHUOK**

*Client Diagnostic Questionnaire - Short Screener 2/1/01*

1. ***Kambi/ Migao*:__________________________ *2. Jatim Nonro ________________________***____________
2. ***Tarik Makawuono* :**_____ /_____/_____***4. Namba Mar Jachiwre*:**|____|____|____|____|____|____|____|____|

Odiechieng / Dwe / Higa

1. ***Nying Jayud Kony kata Nukta Mag nying*_______________________________________________________**

***Instructions to interviewer:***

This questionnaire is designed to facilitate the recognition of the most common mental health problems found in HIV/AIDS primary care or other service settings: mood, anxiety, alcohol and drug abuse, PTSD and thought disorder. Since the questionnaire relies on respondent self-report, definitive diagnoses must be verified by a clinician, taking into account how well the client understood the questions in the ques-tionnaire, as well as other relevant information from family, client records, or other sources.

- 1. Interviewer instructions are printed in bold italics. Questions that you ask or statements that you make to the client are printed in plain type. Read questions as written. Additional probes may be used to ensure client understanding of the question or explore ambiguous answers.
  2. For anything other than a “yes/no” answer, read the answer categories. The interviewer may need to assist the client in answering within the categories given. Never choose an answer category based on what you think the client means by their spoken response.
  3. Be sure that the client is reporting symptoms experienced within the specified time period: past 4 weeks, past 6 months, or in some instances, past 30 days.
  4. Within each module, proceed sequentially from question to question unless instructed either to skip to another question or to go to the next page.
  5. At the end of each diagnostic module is a shaded area with instructions for scoring Positive Screen for each disorder. Scoring can be done by the interviewer or left for office use only.
  6. A Summary Sheet is provided to record “positive screen” or “positive for syndrome” in the spaces pro-vided for each diagnostic module. If no positive screen in any module, indicate in the space provided on the top of the summary sheet.
  7. Space is also provided for interviewer observations and comments. Interviewer should write as detailed as possible description of positive answers to questions especially on psychosis screen. Where known, additional information that may account for symptoms (e.g. medical condition) or history of prior episodes or treatment should be indicated.
  8. *If Client indicates current suicidal feelings or becomes emotionally upset or agitated during interview, please follow agency protocol for contacting your supervisor****.***

The CDQ is based on the PHQ which was developed by Robert L Spitzer, MD, Janet B W Williams, DSW, Kurt Kroenke, MD, et al, and is a modification of the PRIME-MD, which was developed with an unrestricted educational grant from Pfizer, Inc. Adaptation for use by SPNS/ HOPWAProgram Projects by Angela Aidala, PhD and Jennifer Havens, MD with the assistance of Jeffrey Johnson, PhD, Peter Walsh, MD, Cevdet Tosyali, MD, Ezra Susser, MD, and Sally Dodds, PhD, LCSW. For information about using this instrument contact Angela Aidala, PhD, Columbia School of Public Health, 600 W 168th, New York, NY 10032. Phone: (212) 305-7023, email:aaa1@columbia.edu


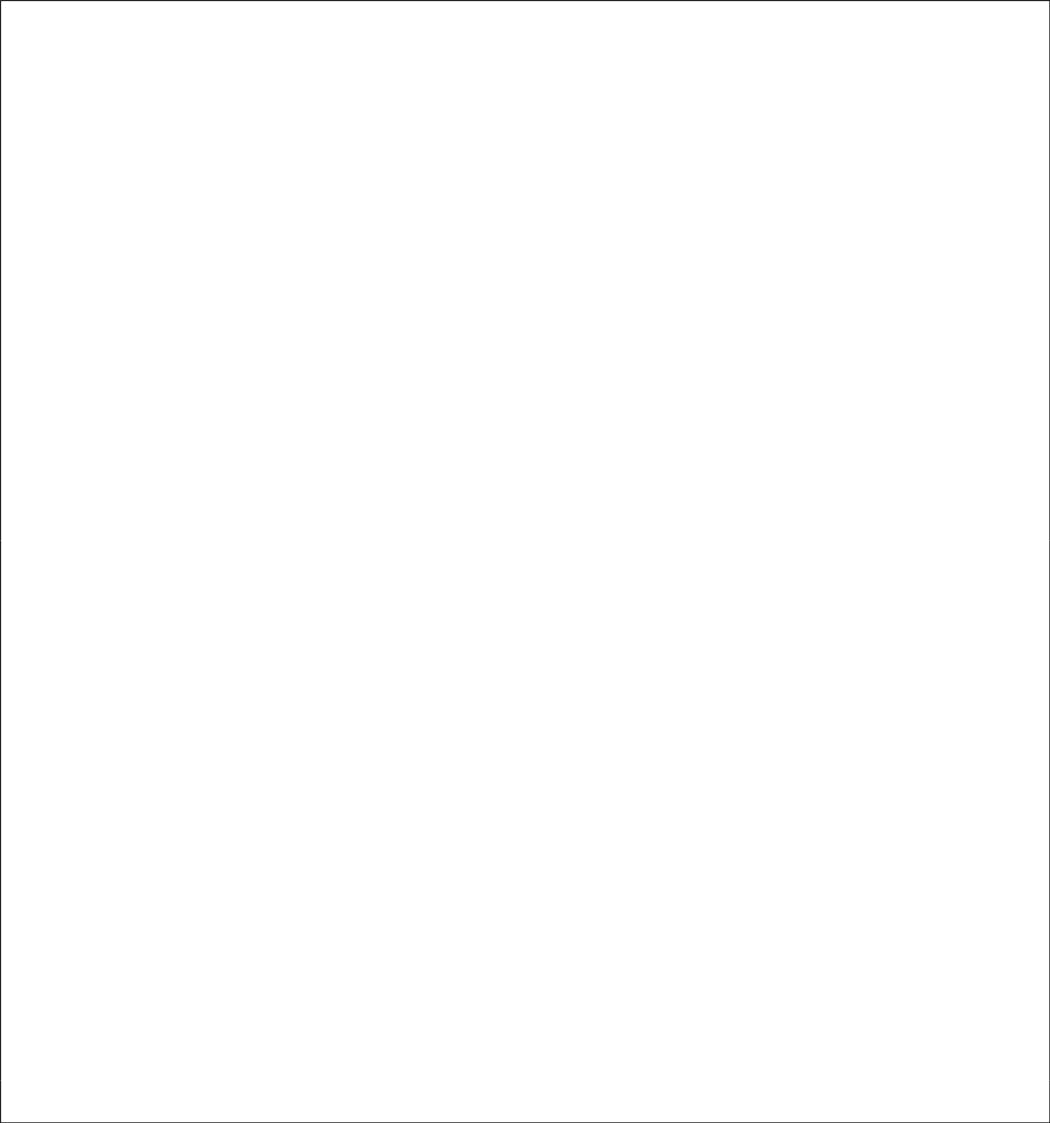


CDQ1

***Wach mokuongo mar Jachiwre.***

Penjogi biro konyowa winjo malong’o chandruok ma inyalo bedogo.. Wapenjo ji duto penjogi mondo wang’e kit konyo kata sir mwanyalo miyi. Yie item duoko penjo ka penjo. Duoko duto te gin maling’ling.

## Ko ong’I eyo machiek

1.Kiparo kuom dweche auchiel mosekalo, mano kuom kinde machalo kaka sanie________ (tarik ma ipimego en dweche 6 motelone nonro) , Ere kaka gik moko osebedo kadhi engimani kaluore gi pachi kata kaka iwinjo ? Bende nitiere kinde moro amora mane ikuyo ahinya kata ne in gi paro mang’eny ? To bende nitie kinde mane in gi kiondko, luoro, kata kibaji kuom gik moko? Bende ne nitiere kinde mane irikni ahinya kata in malo ma ok inyal dok chien?

1. Bende gimoro ne otimoreni ekindeno mane otenore gi kaka ne iwinjo (kata timo) kamano (kuyo, kiondko, bedo malo gi mamoko… refer to symtoms) ? Gimoro amora mane tek kata mane chando chunyi?
2. Eidweche auchiel mosekalo bende ne iwuoyo gi ng’ato ang’ata kuom chandruok mar paro, dendi, kata kaka iwinjo, kata kaka itimori? Ka Ee, Ni iwuoyo gi ng’awa? (Non ane) Bende ne iwuoyo gi jalony kaka laktar kata jahocho? Ang’o mane giwacho kuom mano?

***Interviewer:*** *If client describes symptoms or treatment history, let him/her know that you will be talking about this in* *more detail later in the interview. All screening and appropriate symptom questions must be asked even though topic was discussed in overview. Confirm answers already known.*

CDQ2

**Koro penjo moko ewi pachi, mor kata kuyo kod kaka iwiwnjo. Edwe achiel mokalo (jumbe 4 mokalo) bendenitiere esa mane**...

| Onge, | Odiechienge | Mokalo | Madirom |
| --- | --- | --- | --- |
| Kata | Mang’eny | Nus mar | pipile |
| matin |  | odiechienge |  |

1. Ne ikuyo,iwinjo ka chunyi ool, in gi paro mang’eny, kata ionge gi geno? ***KA EE,*** En mang’eny marom nade mane iwinjo kamano?. . . . . . . . . . .

2. Ne in gi gombo matin kata mor mar timo gik moko? ***KA EE*,**

Ne en mangen’y marom nade mane iwimje kamano? . . . . . . . . . . . . . . . . . . . . . . . . . . . . . . . . . . . . . . . . . . . . . .


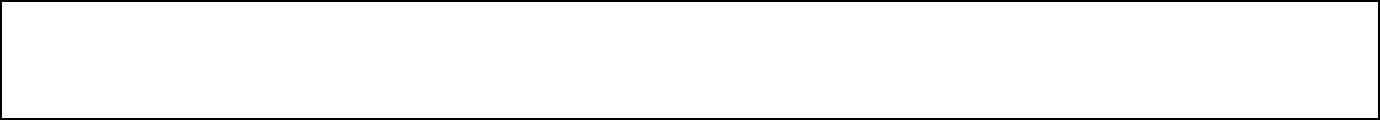


❏  ❏ ❏ ❏

❏  ❏ ❏ ❏

***If client answers “No, Not at all” to both questions, go to next page***

3. En karang’o mane ichake winjo kamano ? (machiegni ahinya)? . . ._________________________

4. Ne obudho maromo nade- ne obudho madirom wige ariyo?. . . . . . . . . . . . . . . . . . . . . ❏ Ooyo ❏ Ee

**Ekindeno, en mang’eny marom nade mane (be isebedo) gi chandruok mar:**

| Onge, | Odiechienge | Mokalo | Madirom |
| --- | --- | --- | --- |
| Kata | mangeny | Nus mar | pilepile |
| matin |  | odiechienge |  |

5. Chandruok dwaro chako nindo, kata nindo ahinya? Kata nindo mang’eny? . . .. . . . . . . . . . . . . . ❏

6. Winjo ki iol kata bedo gi teko matin? . … . . . . . . . . . . . . . . . . . . . . . . . . . . . . . . . . . . . . .❏

7. Dhok marach mar chiemo? Kata chiemo mang’eny? . .. . . . . . . . . . . . . . . . . . . . . . . . . . . ❏

8. Chari iwuon- kata ni irem kata ni igoyo joodi piny?.. . ……………………………….. ❏

9. Pek mar ketopachi egikmoko, kaka somogaset, neno wangjowi, winjo kaka ng’ato tayi kata luwo kaka mbaka dhi,? ❏

1. Wuotho kata wuoyo mosahinya majomoko nyalo bedoni ne ofwenyo? Kata ataro marmagi- mbwakni kata koso dembruok ma iwuotho mang’eny ma opogore kod pile?...... . . …………… . . ❏

11. Ne in gi paro ni ber ka ithoo kata paro mar inyori iwuon eyo moro . . . . . . .. . . . . . . . . . . . ❏


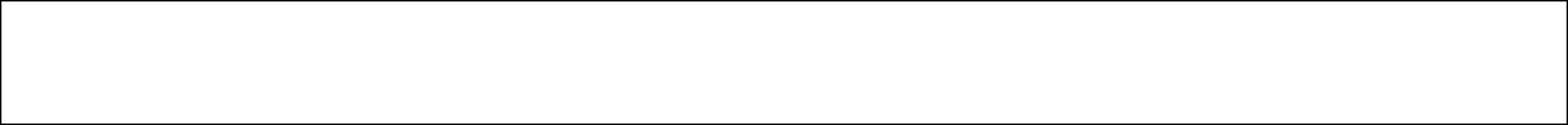


❏  ❏ ❏

❏  ❏ ❏

❏ ❏ ❏

❏  ❏ ❏

❏  ❏ ❏

❏  ❏ ❏

❏  ❏ ❏

Maj Dep Syn if 2 weeks (Q4) is “yes” (AND) answer to question 1 or 2 is shaded (AND) 5+ of answers to any of Q. 1, 2, 5 - 11 are shaded; Other Dep Syn same but only 2+ of the answers to Q. 1, 2, 5 - 11 are shaded

CDQ3

**CDQ4**

**Koro penjo moko ewi kiondko...**

**EE** **OOYO**

1. Kuom jumbe 4 mosekalo, bende kiondko osemonji– winjo

apoya luoro kata buok?.. . . . . . . . . . . . . . . . . . . . . . . . . . . . . . . . . . . . . . . . . . . ……… ❏
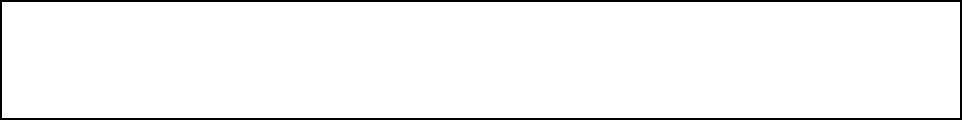
❏

***If client answers “NO” go to next page***

2. Be ma osega timore ekinde mokalo? . . . . . . . . . . . . . . . . . . . . . . . . . . . . . . . . . . . . . . ❏ ❏

1. Bende moko kuom monj gi ose bironi apoya nono- eseche maok ok ichano ni inyalo bedo gi kiondko kata bedo maonge kwe?

|  |  | . . . . . . . . . . . . . . . . . . . . . . . . . . . . . . . . . . . . . . . . . . . . . . . . . . . | ❏ | ❏ |
| --- | --- | --- | --- | --- |
| 4. Bende monj gi chando chunyi ahinya? Bende chunyi chandore ni inyalo yudo monj machielo? | | |  |  |
|  | . . . . . . . . . . . . . . . . . . . . . . . . . . . . . . . . . . . . . . . . . . . . . . . . | | ❏ | ❏ |

**Parane monjo mogik mane in go marach ahinya..**

| 5. | Be ne iyweyo gi chandruok? . . . . . . . . . . . . . . . . . . . . . . . . . . . . . . . . . . . . . . . . . . . . . . . . | ❏ | ❏ |
| --- | --- | --- | --- |
| 6. | Be chunyi ne oridore,gwecho matek, kata ochikore?. . . . . . . . . . . . . . . . . . . . | ❏ | ❏ |
| 7. | Be ne intiere gi rem ekori kata tuo mar chuny maguecho matek? . . . . . . . . . . . . . . | ❏ | ❏ |
| 8. | Bende ne ichwero luya? . . . . . . . . . . . . . . . . . . . . . . . . . . . . . . . . . . . . . . . . . . . . . . . . . . . | ❏ | ❏ |
| 9. | Bende ne iwinjo ka gima gimoro deyi? . . . . . . . . . . . . . . . . . . . . . . . . . . . . . . . . . | ❏ | ❏ |
| 10. | Be ni iwinjo liet edendi kata koyo? . . . . . . . . . . . . . . . . . . . . . . . . . . . . . . . . . . . . . . . | ❏ | ❏ |
| 11. | Be chunyi no olewi kata iyi omuori, kata winjo ka gima idhi diewo? |  |  |
|  | . . . . . . . . . . . . . . . . . . . . . . . . . . . . | ❏ | ❏ |
| 12. | Bende ne in gi kawirawira, bedo ma ok ongirore, kata winjo ka muchi ng’adore?. . . . . . . . . …………………………. . . . . . . . . . | ❏ | ❏ |
| 13. | Bende dendi ne chuore (pino gi sindene) kata otho |  |  |
|  | thudhno?. ………………………. . . . . . . . . . . . . . . . . . . . . . . . . . . . . . . . . . . . . . . . . | .❏.. . . . . . . . . . . | ❏ |
| 14. | Bende ni itetni kata kirni? . . . . . . . . . . . . . . . . . . . . . . . . . . . . . . . . . . . . . . . . . . . . . . . . . . | ❏ | ❏ |
| 15. | Bende ne in giluoro ni itho? . . . . . . . . . . . . . . . . . . . . . . . . . . . . . . . . . . . . . . . . . . . | ❏ | ❏ |


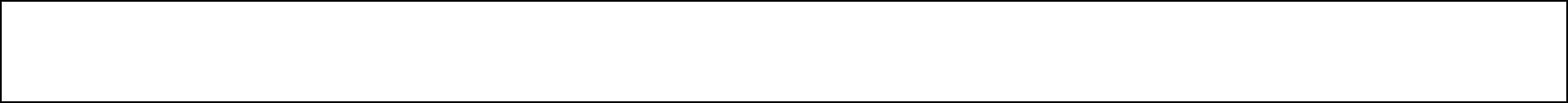


Pan Syn if answers to Q. 1,2,3 and 4 are ‘Yes’ (AND) 4+ symptoms during an attack (Q. 5-15)

CDQ4

**CDQ5**

**Ejumbe 4 mokalo, en mang’eny marom nade ma isebedo gi chandruok mar:**

|  | Onge, | Odiechienge | Mokalo | Machiegeni |
| --- | --- | --- | --- | --- |
|  | Kata matin | Mang’eny | Nus | Odiechieng ko diechieng |
|  |  |  | Odiechienge |  |
| 1. Winjo kibaji,kiondko,winjo ka gima in egiko, kata bedo gi chumy machandore mangeny kuom gik mopogore? |  |  |  |  |
|  |  |  |  |
| . . . . . . . . . . . . . . . . . . . . . . . . . . . . . . . . . . . . . . . . . . . . . . . . . . . . . . . . . . . . . . . . . . | ❏ | ❏ | ❏ | ❏ |
|  |  |  |  |  |


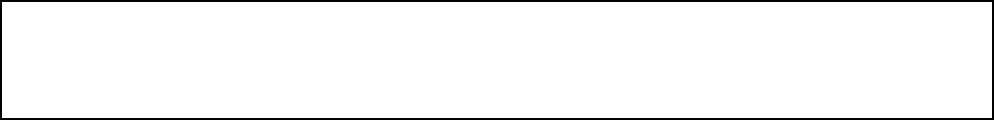


***If client answers “Not at all” go to next page***

| 2. | Dang’ni ma ok inyal dembori?.......................... | ❏ | ❏ | ❏ | ❏ |
| --- | --- | --- | --- | --- | --- |
| 3. | Bedo gi olo eyo mayot? . . . . . . . . . . . . . . . . . . . . . . . . . . . . . . . . . . . . . . . . . . . . . . . . . | ❏ | ❏ | ❏ | ❏ |
| 4. | Jony,rem kata lit mag leche del? . . . . . . . . . . . . . . . . . . . . . . . . . . . . . . . . . . . . . . . . . . | ❏ | ❏ | ❏ | ❏ |
| 5. | Chandruok chako nindo kata nindo matut? . . . . . . . . . . . . . . . . . . . . . . . . . . . . . . . . . . | ❏ | ❏ | ❏ | ❏ |
| 6.. | Pek mar keto pachi egik moko, kaka somo gaset, neno wang jowi, winjo ka ng’ato tayi kata luwo kaka mbaka dhi,?................................................................... |  |  |  |  |
|  |  | ❏ | ❏ | ❏ | ❏ |
|  |  |  |  |  |  |
| 7. | Ich mawan’g kata chuny machuanyore piyo? . . . . . . . . . . . . . . . . . . . . . . . . . . . . . . . . . . . . . . . . | ❏ | ❏ | ❏ | ❏ |


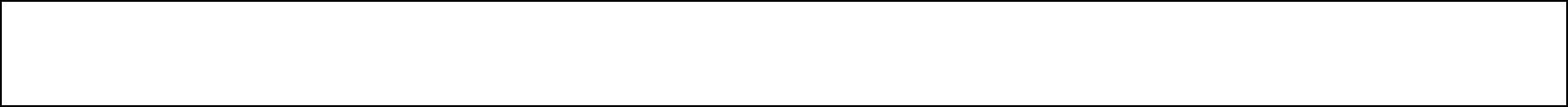


Other Anx Syn if answer to Q. 1 is shaded (AND) 3+ answers to Q. 2-7 are shaded.

CDQ5

**CDQ6**

**Penjo moluwogi gin kuom math kong’o kod tiyo gi yedhe mer mamoko. Wapenjo penjogi kaka weche mag ngima ji te. Gik moko duto minyisa gin siri matut kendo oriti**.

1. Edweche auchiel mosekalo, en mang’eny marom nade ma isemadhe konge kaka bia, amuna kata kon’go makech?

|  | Matin ne |  |  |  |  |
| --- | --- | --- | --- | --- | --- |
| Podi | Dichiel edwe | Dwe ka dwe | Juma ka Juma | Nyadidek ejuma | Pilepile |
|  |  |  |  |  |  |
| ❏ | ❏ | ❏ | ❏ | ❏ | ❏ |
|  |  |  |  |  |  |


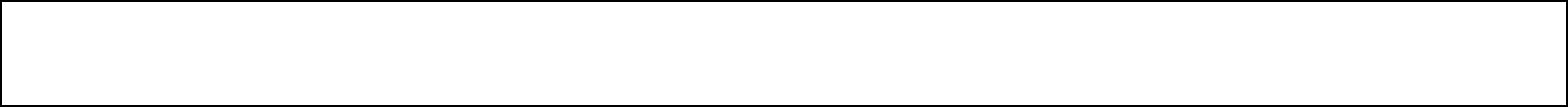


***If client never drinks alcohol, go to last alcohol question - Q.13 next page***

******Please use the worksheet "Calculation of Number of Drinks Consumed" for Q.2******

2. Gin math adi ma imadhoga e odiechienge mi metho?

| Achiel | Ariyo | Adek | Ang’wen | Abich |
| --- | --- | --- | --- | --- |
| ❏ | ❏ | ❏ | ❏ | ❏ |

**Bende moro amora kuom gigi osetimi mang’eny ne dichiel edweche 6, mano chakre ( ) nyaka kawuono?**

***(fill in date 6 mo prior to interview)***

**EE** **OOYO**

3. Ne imadho kong’o kata bed ni laktar ne ong’ado ni rieko ni iwe

Metho kaluore gi chandruok engimani? . . . . . . . . . . . . . . . . . . . . ❏ ❏

1. Ne imadho kong’o, ne imer, iwe tich nikech jony bang metho, dhi skul, kata rito nyithindo kata timo tije mamoko?

|  | . . . . . . . . . . . . . . . . . . . . . . . . . . . . | ❏ | ❏ |
| --- | --- | --- | --- |
| 5. | Ne ok idhi kata ilewo ne gima ochuno nikech ne imetho kata ne ijony |  |  |
|  | bang metho? . . . . . . . . . . . . . . . . . . . . . . . . . . . . . . . . . . . . . . . . . . . . . . . . . | ❏ | ❏ |
| 6. | Ne in kod chandruok mar winjori gi ji mamoko ekinde mane imetho? |  |  |
|  | . . . . . . . . . . . . . . . . . . . . . . . . . . . . . . . . . . . . . . . . . . . . . . . . . . . . . . . . . . . . . . . . . . | ❏ | ❏ |
| 7. | Ne iriembo nyamburko (nyamburko matin, lori, apiko) kata tiyo gi masinde madongo bang madho kong’o mathoth kata bang math mang’eny ahinya? |  |  |
|  |  | ❏ | ❏ |


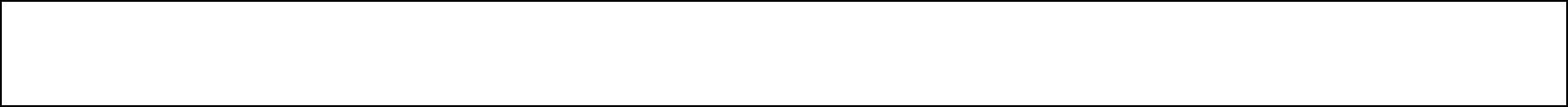


Mokalo Abich

❏

Alc Abu if 1+ answers to Q. 3-7 are Yes (OR) 5+ drinks a day weekly or more often

CDQ6

CDQ7

Endalo 30 MOSEKALO**,** mano**,** kaka ekindeni **(_______________** **) ...**

*(month prior to interview)*

8. Gin odiechienge adi mane imadho gimoro amora manigi kong’o eiye ? |___|___|


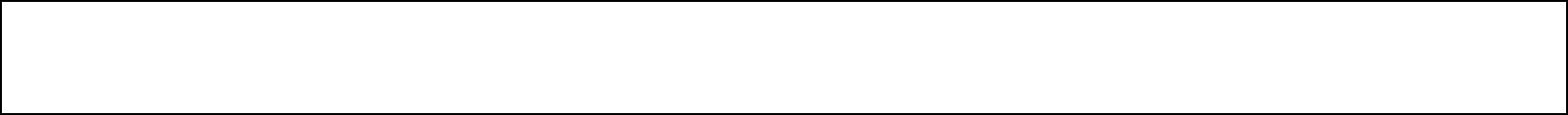


***If client never drank alcohol past 30 days, go to last alcohol question - Q.13 below***

E odiechienge 30 mokalo...

|  |  | **EE** | | **OOYO** |
| --- | --- | --- | --- | --- |
|  |  |  | |  |
| 9. | Bende isebedo giparo ni onego iduok chien kaka imadho kong’o? . . . . . . . . | ❏ |  | ❏ |
| 10. | Bende ng’ato osewuoye ewach methoni? . . .. . . . . . . . . . . . . . . . . . . . . . . . . . | ❏ |  | ❏ |
| 11. | Bende isewinjo kagima ikoso kata iyi osewang’ kuom mer mari? .. . . . . . . . . . . . . | ❏ |  | ❏ |
| 12. | Bende nitie odiechieng achiel mane imadho chupni abich kata mang’eny |  |  |  |
|  | Mokalo mar bia, amuna kata kong’o makech?. . . . . . . . . . . . . . . . . . . . . . . . . . . . . . . . . . . . . . . . . | ❏ |  | ❏ |
| ***ASK EVERYONE*** | |  |  |  |
|  |  |  |
| 13. | Bende ne in kata ng’at ang’ata machiegni kodi opare ni in kod chandruok |  |  |  |
|  | mar kong’o? . . . . . . . . . . . . . . . . . . . . . . . . . . . . . . . . . . . . . . . . . . . . . . . . . . . . . . . . . . . . . . . . . . .. . . | ❏ Ee | | ❏ Ooyo |


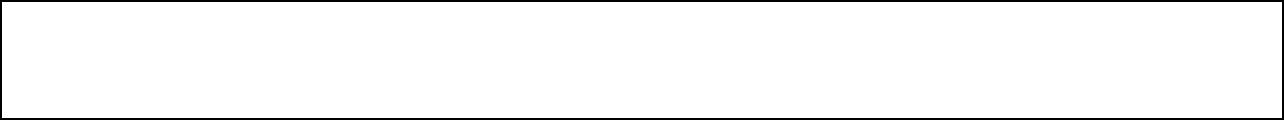


Alc Abu 30 day if 2+ answers to questions 9-12 are YES

CDQ7

**CDQ8**

**Koro endi nitie penjo moko ewi tiyo kod yedhe ma mero ji.** (*Remind client of confidentiality*)

## Ng’e ni gik moko duto ma inyisa gin maling’ling makende kendo okan maber.

Bende isetiyo kod moro amora kuom yedhe gi, kata nyadichiel...


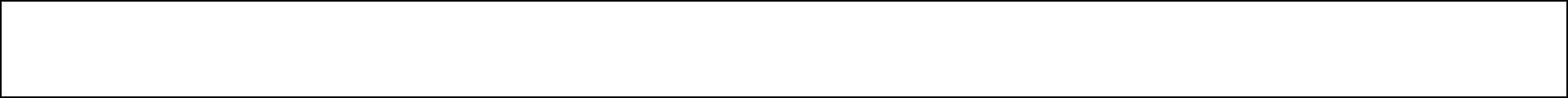


***GO DOWN THE ENTIRE LIST, then go back and for any drug used, ask about use past six months***

|  |  |  |  | *Ka EE ne yath moro amora penji*: | | |  |  |  |
| --- | --- | --- | --- | --- | --- | --- | --- | --- | --- |
|  |  | Be isega tiyo gi |  | Edweche auchiel mosekalo, didi mane itiyo kod (yath)? | | | | | |
|  |  |  |  |  |  |  |  |  |  |
|  |  |  |  |  | Matin ne |  |  |  |  |
|  |  |  |  |  | dichiel |  |  | Nyadidek |  |
|  |  | Ee | Ooyo | Podi | edwe | Dwe ka dwe | Juma ka Juma | ejuma | Odiechieng’kodiechieng’ |
| 1. | Njaga ( Nyasore,hashish,cannabis | | ❏ | ❏ | ❏ | ❏ | ❏ | ❏ | ❏ |
|  | weed, obwa, bangi, ganja, . ❏ | |
|  | sensi, boza, buya,mbom,) |  |  |  |  |  |  |  |  |
| 2. | Cocaine………………………. ❏ | | ❏ | ❏ | ❏ | ❏ | ❏ | ❏ | ❏ |
| 3. | Crack, freebase . . . . . . . . . . . . | ❏ | ❏ | ❏ | ❏ | ❏ | ❏ | ❏ | ❏ |
| 4. | Heroin, brown sugar, white cap, | | ❏ | ❏ | ❏ | ❏ | ❏ | ❏ | ❏ |
|  | white crest, unga. . | ❏ |

1. Methadone (Yath mathiedho weyo yedhe ma meroji ka ok ondikni kata mangen’y makalo kaka laktar ondikoni)

| ?. . . . . . . . | ❏ | ❏ | ❏ | ❏ | ❏ | ❏ | ❏ | ❏ |
| --- | --- | --- | --- | --- | --- | --- | --- | --- |

1. Sedatives or downers (valium, stilnox, cough syrup) without a pre-scription or more than a

| doctor told you to(Yath makweyi/myi nindo ka ok ondikni gi laktar kata mangeny mang’eny mokalo kaka laktar onyisi). . . . . . . . . . | ….❏ | ❏ | ❏ | ❏ | ❏ | ❏ | ❏ | ❏ |
| --- | --- | --- | --- | --- | --- | --- | --- | --- |

1. Stimulants (methamphetamine uppers, speed, ice) without a

|  | prescription or more than a | | |  | ❏ | ❏ | ❏ | ❏ | ❏ | ❏ | ❏ |
| --- | --- | --- | --- | --- | --- | --- | --- | --- | --- | --- | --- |
|  | doctor told you to(Yath mamedi teko/miyo ok inind ka ok ondikni gi laktar kata mangen’y mokalo kaka laktar ondiko . . . . . . . . . . ❏ | | | |
| 8. | Hallucinogens (PCP, | | |  |  |  |  |  |  |  |  |
|  | angel dust, ecstasy, | | |  |  |  |  |  |  |  |  |
|  | mushrooms, LSD(Yath mamiyo ineno gik magala gala /kata ibedo gi mor ma okalo tong’ . . . . . . . . . . | | | ❏ | ❏ | ❏ | ❏ | ❏ | ❏ | ❏ | ❏ |
| 9. | Sniffed or inhaled | | |  |  |  |  |  |  |  |  |
|  | anything to get high | | |  |  |  |  |  |  |  |  |
|  | (sprays, glue) (Ifito ,Ming’weyo kata iywayo mondo imer). . . | | | ❏ | ❏ | ❏ | ❏ | ❏ | ❏ | ❏ | ❏ |
| 10Mamoko(**see Appendix 2**) | | | | ❏ | ❏ | ❏ | ❏ | ❏ | ❏ | ❏ | ❏ |
|  |  |  |  |  |  |  |  |  |  |  |  |

(Ler: ________________)

*IF EVER USED ANY DRUG:*

11. Bende osechuoyi yath maloko paro kata kete (epien del) gi sindan, kata dichiel??

*IF EVER USED NEEDLE:*

1. Bende osechuoyi kata keto yath maloko paro (epien del) gi sindan, kata dichiel edweche auchiel mokalo? Ka ee, yath mane?, ?_____________________


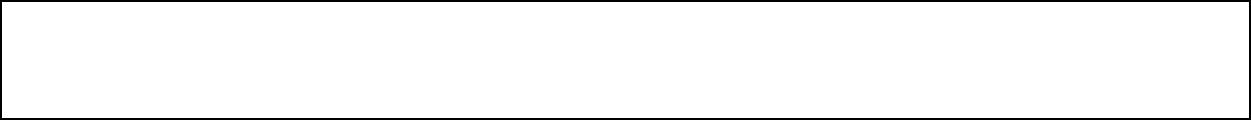


***If No Drug Use IN 6 MONTHS go to PAGE 11 Trauma***

CDQ8

*Ee* *Ooyo*

❏  ❏

❏  ❏


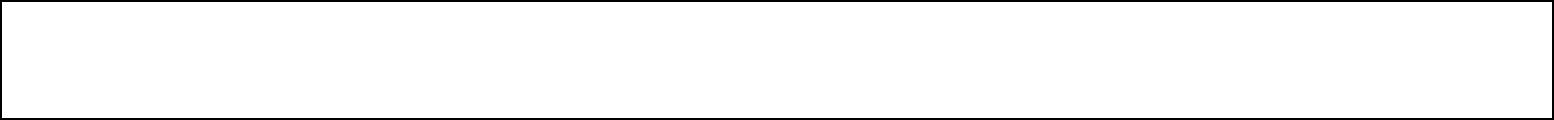
***Ask all clients who have used any drug in past 6 mos***

**Bende gimoro amora kuom gigi osetimoreni mang’eny ne nyadichiel edweche 6 mokalo, mano chakre ( ) nyaka kawuono?**

fill in date 6 mo prior to interview

**EE** **OYOO**

13. Ne itiyo kod yedhe maloko paro kata bed ni laktar ne osenyisi ni iwe tiyogo

nikech chandruok mar ngimani? . . . . . . . . . . . . . . . . . . . . . . . . . ❏ ❏

14.Ne itiyo gi yedhe mameroji, ne imer, iwe tich nikech jony bang metho, dhi skul, kata rito nyithindo kata timo tije mamoko?

|  | mamoko? . . . . . . . . . . . . . . . . . . . . . . . . . . . . . . . . . . . . . . . . . . . . . . . . . . . . . . . . . . . . | ❏ | ❏ |
| --- | --- | --- | --- |
| 15. | Ne ok idhi kata ilewo ne gima ochuno nikech ne imetho kata ne ijony |  |  |
|  | bang metho? . . . . . . . . . . . . . . . . . . . . . . . . . . . . . . . . . . . . . . . . . . . | ❏ | ❏ |
| 16. | Ne in kod chandruok mar winjori gi ji ekinde mane itiyo |  |  |
|  | gi yedhe mameroji? . . . . . . . . . . . . . . . . . . . . . . . . . . . . . . . . . . . . . . . . . . . . . . . . . . . . . . . . . . . . . . . . . | ❏ | ❏ |
| 17. | Ne iriembo nyamburko bang tiyo gi yethe mameroji? . . . . . . . .. . . . . . . . . . . . . . . . . . . | ❏ | ❏ |
| 18. | Ne ibedo gi chandruok mar chik nikech tiyo kod yethe mameroji…(tudruok gi obila, |  |  |
|  | oteri edoo/ng’ato odonjoni, kata chandruok moro amora gi chik)? ........ | ❏ | ❏ |


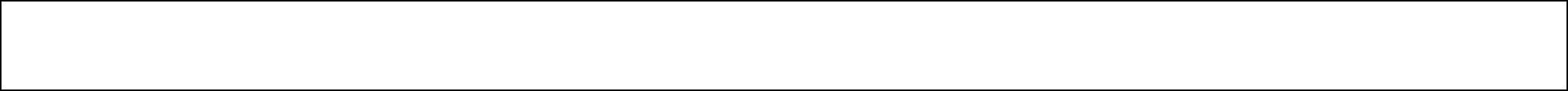


DRUG ABU if 1+ answers to Q 12 - Q 17 are Yes (OR) Heroin, Coke/Crack or Methamphetamine 3+ per week

CDQ9

| E odiechienge 30 MOKALO, mano, chakre sechegi e **(** | | |  | **) ...** |
| --- | --- | --- | --- | --- |
|  |  | *month prior to interview* | | |
| Gin odiechienge adi mane itiyo kod... | |  |  |  |
| 14. | Marijuana (Nyasore) . . . . . . . . . .. . . . . . . . . . . . . . | |___|___| |  |  |
| 15. | Cocaine (Yath ma mero ji mar Mogo.) . . . . . . | |___|___| |  |  |
| 16. | Crack ( Yath ma mero ji chenga) . . . . . . . . . . . | |___|___| |  |  |
| 17. | Heroin or speedball( (Yadh chenga gi mogo | |___|___| |  |  |
| 18. | Sedatives, Downers . (Yath makweyi/miyi nindo). | |___|___| |  |  |
| 19. | Stimulants, Uppers(Yath mamedi teko/miyo ok inind | |___|___| |  |  |
| 20. | Hallucinogens(Yath mamiyo ineno gik magalagala). . | |___|___| |  |  |
| 21. | Inhalants(Yedhe ma iywayo to meroji). . . . . . . . . . . . | |___|___| |  |  |


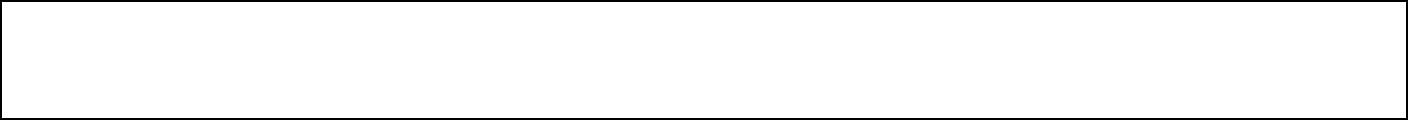


***If client never used any drug past 30 days, go to next page***

E odiechienge 30 mokalo….

|  |  | **YES** | **NO** |
| --- | --- | --- | --- |
| 22. Bende isegaparo mar duoko chien tiyo gi yedhe mameroji? | |  |  |
|  |  | ❏ | ❏ |
| 23. | Bende ng’ato oseng’ur ewi tiyoni gi yedhe mameroji? . . . . . . . . . . . . . . . . . . . . . . | ❏ | ❏ |
| 24. | Bende isewinjo wich kuot kata bedo gi ich wang’ kuom tiyo gi yedhe mameroji? | ❏ | ❏ |
| 25. Bende isetiyo gi yath mamero ji moro amora di 3 kata ndalo mang’eny ejuma kata kinde ka kinde?. . | | ❏ | ❏ |
|  |  |  |  |


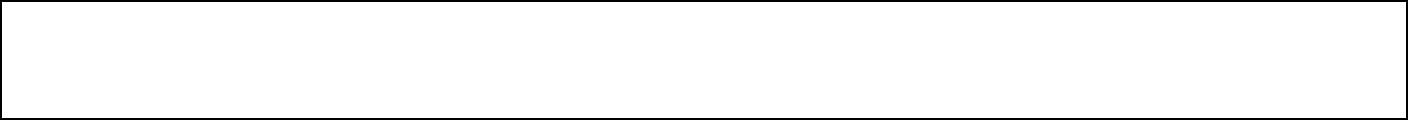


Dru Abu 30 day if 2+ answers to questions 22-25 are Yes

CDQ10

*ASK EVERYONE*

**Koro penjo moko ewi gik maricho ahinya kata gik mabuogi ma dipo ni ne osetimoreni.**

Ji kinde ka kinde kadhe gik mabuogo ngima. Ma nyiso ni gik maricho ahinya, gigo mabuogo ji. Adhi somo moko kuom gik manyalo timore ne ji. Yie inyisa ka ma osetimoreni...

|  |  | EE | OOY O |
| --- | --- | --- | --- |
| 1. | Masira marach (edala,nyamburko, apiko) kata mach edala kata ekar tiji. . . | . . ❏... . . . . . . . . . . . . . | .❏... . . . . |
| 2. | Masira mar piny maok nyal geng’ kaka hayiti, |  |  |
|  | yieng’ni mar piny maduong, yamo malich, oula, kata masirni machal kama . . . . . . | . .❏... . . . . . . . . | ❏ |
| 3. | Yudori e lueny . . . . . . . . . . . . . . . . . . . . . . . . . . . . . . . . . . . . . . . . . . . . . | ❏ | ❏ |
| 4. | Gocho kata sand gi jaherani kiduong. . . . . . . . . . . . . . . | ❏ | ❏ |
| 5. | Gocho kata sand kata kwali eyor angenge kiduong gi ng’at | ❏ | ❏ |
|  | mopogore gi jaherani . . . . . . . . . . . . . . . . . . . . . . . . . . . . . . . . . . . . . . . . . . . . . . . . . . . . . |
| 6. | Gocho kata sand kane in nyathi. . . . . . . . . . . . . . . . . . . . . . . . . . . . . . . . . . . . . . . . . . . | ❏ | ❏ |
| 7. | Neno ka ji gore kata hinyo jo wetegi eodu |  |  |
|  | ekinde mane idongo . . . . . . . . . . . . . . . . . . . . . . . . . . . . . . . . . . . . . . . . . . . . . . | ❏ | ❏ |
| 8. | Achune mar terruok kata terruok kodi gi thuon ka iduong.. . . . . . . . . . . . . . . . | ❏ | ❏ |
| 9. | Achune mar terruok kata terruok kodi gi thuon ki inyathi . . . . . . . . . . . . . . . . . . . . | ❏ | ❏ |
| 10. | Neno ka ng’ato igoyo kata isando . . . . . . . . . . . . . . . . . . . . . . . . . . | ❏ | ❏ |
| 11. Neno ka ihinyo ng’ato marach kata ka inego eyor ang’enge . . . . . . . . . . . . | | ❏ | ❏ |
| 12. Lalo nyathini ko okalo etho. . . . . . . . . . . . . . . . . . . . . . . . . . . . . . . . . | | ❏ | ❏ |
| 13. | Ich wuok.. . . . . . . . . . . . . . . . . . . . . . . . . . . . . . . . . . . . . . . . . | ❏ | ❏ |

14. Gimoro ma ok ber kata makelo buok madibed ni ne otimoreni?

Ler ane____________________________________

________________________________________________________

________________________________________________________

________________________________________________________


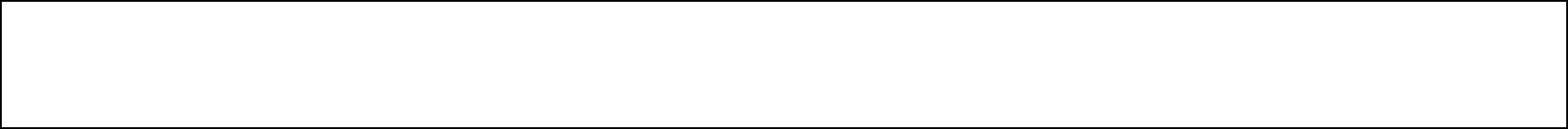


***If client answers “NO” to all questions go to Page 13, PSY***

***If client answers “YES” to one or more questions go to the NEXT PAGE***

CDQ11


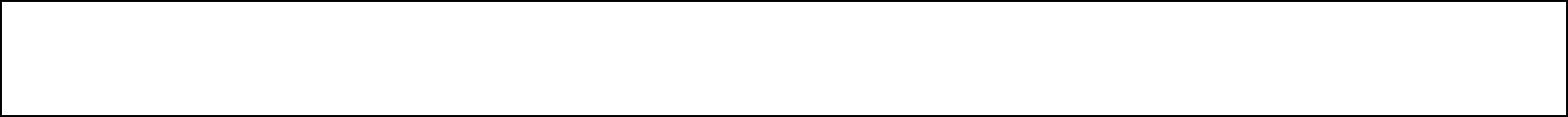
***If client answers “YES” to ONLY ONE event listed on the previous page, Ask Q. 1A***

1A. Isenyisa kuom samane______ (yang gima ne otimore).

Daher mar penji matut matin ewi gima ne otimore ni. . . . . . . . . ***skip to Q.2***

***
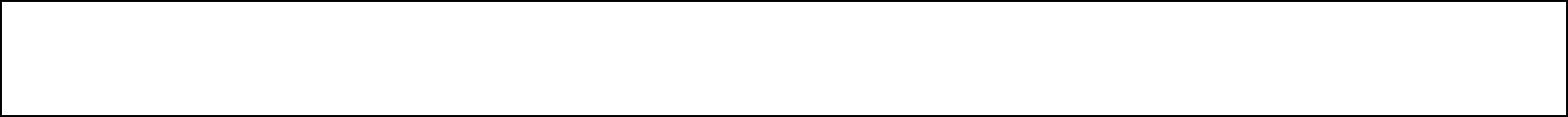
***

***If client answers “YES” to MORE THAN ONE event on the previous page, Ask Q. 1 B***

1B. Isenyisa gik mang’eny mane otimoreni. En mane kuom gigi mane rach ahinya kata mane obuogi ahinya? ? _________________________ *(ler gima ne otimore kata gik moloure ma jayud kony owacho)*

**Daher mar penji mathoth matin kuom gima ne otimoreni (kaka negi timore)...**

2. Ne ibuok machal nade...

|  | ❏ | ❏ | ❏ | ❏ |  | ❏ |
| --- | --- | --- | --- | --- | --- | --- |
|  | Onge Kata matin | Mana matin | Marach | Marach ahinya |  | Buok but tho |
| Edweche auchiel mokalo**...** | | |  |  |  |  |
|  |  |  |  |  | **EE** | **OOYO** |
| 3. | Isiko ipare kata ka ok idwar? . . . . . . . . | | | . . . . . . . | ❏ | ❏ |
| 4. | Bende ibedo gilek mabuogi kuome? . . . . . . . . . | | . . . . . . . . . . . . . . . . . . . . . . . | . . . . . . . | ❏ | ❏ |
| 5. | Be gik maparoni gima ne otimore mayi mor kata chando chunyi? . . . . . . . . . | | | | . .❏.. . . . . . | . . . . ❏ |
| 6. | Bende iparoga- paro mapore ni gicha biro | | |  |  |  |
|  | timore kendo? . . . . . . . . . . . | | . . . . . . . . . . . . . . . . . . . . . . . | . . . . . . . | ❏ | ❏ |
| 7. | Bende ibedo kod paro mang’eny ni onyalo timore kendo?...................................................................... | | . |  | ❏ | ❏ |
| 8. | Be igeng’o gik maparoni ewi gima ne otimoreno?. . | | . . . . . . . . . . . . . . . . . . . . . . . | . . . . . . . | ❏ | ❏ |
| 9. | Bende seche moko ibedo kod chandruok mar paro gimane | | |  |  |  |
|  | otimore malong’o?. . . . | . . . . . . . . . . . . . . . . . . . . . . . . . . . . . . . . . . . . . . . . . . . . . . . . . . . . . . | | . . . . . . . | ❏ | ❏ |
| 10. Be iwinjo ni in kendi kata bed ni in kod jomamoko, | | | |  |  |  |
|  | kata winjo ni gi pek riuruoki gi ji? | . . . . . . . . . . . . . . . . . . . . . . . . . . . . . . . | . . . . . . . . . . . . . . . . . . . . . . . | . . . . . . . . . | ❏ | ❏ |
| 11. Be iwinjo kalandhidhi kata ni ok in kod gombo motegno | | | |  |  |  |
|  | ne gimoro amora? | . . . . . . . . . . . . . . . . . . . . . . . . . . . . . . . | . . . . . . . . . . . . . . . . . . . . . . . | . . . . . . . | ❏ | ❏ |

12. Bende ibedo gi luoro, ibet kod kiondko, ibuok piyo kata bedo ni ikichori ma

onge gima omiyo? . . . . . . . . . . . . . . . . . . . . . . . . . . . . . . . . . . . . . . . . . . . . . . . . . . . . . . . . . . . . .❏. ❏

****If client answers “YES” to MORE THAN ONE event on the previous page, ask Q.1C in Appendix 3 ****


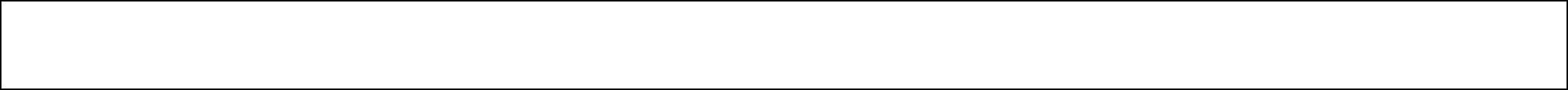


PTS Syn if answer to 2 is “Bad” or worse (AND) 1+ answers to Q 3-6 (AND) 2+ answers to Q.8-11 are YES

CDQ12

**Koro adhi penji ewi chike/yie gi paro ma jomoko nitierego. Jomoko ni kod parogi kod yie bang’ ka gisebedo ka gi madho kong’o kata tiyo gi yedhe ma meroji. Daher penji ka isegabedo gi paro makama EJUMBE 4 (odiechienge 30) mokalo ekinde MA OK isebedo ki imadho kong’o kata tiyo gi yedhe ma meroji.**

|  |  |  |  |  |  | **Mokalo** |
| --- | --- | --- | --- | --- | --- | --- |
|  |  |  |  |  | **Dichiel** | **dichiel** |
| **Ejumbe 4 mokalo, mang’eny marom nade . . .** | | | | **Podi** |  |  |
| 1. Bende isewinjo koko kata duol ma jomoko wacho ni ok gi nyal winjo? . . . . . . . . . . . . . | | | | ❏ | ❏ | ❏ |
|  | ***Ka EE:*** Nyisa ni ang’o mane iwinjo? Ka duol: Ang’o mane duol wacho? Bende duol go ne onyisi ni itim gimoro? Ang’o? Dibed ni duol wuok eyii koso wuok oko? | | |  |  |  |
|  |  | | |  |  |  |
|  |  |  |  |  |  |  |
|  |  |  |  |  |  |  |
|  |  |  |  |  |  |  |
|  |  |  |  |  |  |  |

| 2. Bende isewinjo ni nenitiere jok mane dwa hinyi?.......... **Ka EE:** Gin jomage? Ang’o momiyo gi dwa hinyi? Be luoroni miyo ibedo kod pek wuok edalani kata kama inindega pile?. . . . | | | . . . . . . . ❏ | ❏ | ❏ |
| --- | --- | --- | --- | --- | --- |
|  |  | |  |  |  |
|  |  | |  |  |  |
|  |  |  |  |  |  |
|  |  |  |  |  |  |
|  |  |  |  |  |  |
|  |  |  |  |  |  |

3. Bende isega winjo ni gimoro rach kata gima ok mapile timore e aluora mari kata ni

Gik moko e aluorini lokore? . . . . . . . . . . . . . . . . . . . ._. . . . . . . . . . . . . . .

❏

❏

❏

***Ka EE:*** Be inyalo nyisa ewi mano? Be iwinjo ka gima ji chano chenro maok kare ne in?

Be gik moko nyiso gima duong kata mani gi nengo ne in? Ka kwan kata ranyisi mag ndara kata gima chalo kamano?

| 4. Be iseneno fweny kata neno gik ma jomoko wacho ni ok gi nyal neno? . . ……………. . . . . . . ❏ | | | | ❏ | ❏ |
| --- | --- | --- | --- | --- | --- |
|  | ***Ka EE:*** Nyisa gigo ma iseneno. Be ma timore ekinde ma ineno? | | |  |  |
|  | Otimore kanye? Be ineno ng’at mane otho | | |  |  |
|  | machiegni? |  |  |  |  |
|  |  |  |  |  |  |
|  |  |  |  |  |  |
|  |  |  |  |  |  |

| 5. Be isewinjo ni in kod teko moko ma opogore gi mapile ma joma moko onge go? . | | | . . . . . . . . . . ❏ | ❏ | ❏ |
| --- | --- | --- | --- | --- | --- |
|  | **Ka EE:** Nyisa teko gi. Ere kaka gi pogore gi gik ma ji mamoko nyalo timo? | |  |  |  |
|  | Ere kaka isetiyo gi tekogi? |  |  |  |  |
|  |  |  |  |  |  |
|  |  |  |  |  |  |
|  |  |  |  |  |  |

| 6. Bende isega bedo gi paro ni in kod juogi kata in gi jachien? . . . . . . . . | | | . . . . . . . . . . ❏ | ❏ | ❏ |
| --- | --- | --- | --- | --- | --- |
|  | **Ka EE:** Be inyalo nyisa mathoth kuom wachno? Be ne juogi/jachien omiyo | |  |  |  |
|  | itimo gimoro? To en ang’o? |  |  |  |  |
|  |  |  |  |  |  |
|  |  |  |  |  |  |

CDQ13

|  |  |  |  |  | **Mokalo** |
| --- | --- | --- | --- | --- | --- |
|  |  |  |  | **Nyadichiel** | **dichiel** |
| **Ejumbe 4 mokalo, mang’eny marom nade…** | | | **Podi** |  |  |
| 7. Be isega winjo kagima pachi lal kata gimoro mawuok oko | | |  |  |  |
|  | mayi pachi? . . . . . . . . . . . . . . . . . . . . . . . . . . . . . . . . . . . . . . . . . . . . . . . . . . . . . . . . . . . . . . . . . . . . . | | . . . . . . . . . . . ❏ | ❏ | ❏ |
|  | Ka EE: Ng’a kata ang’o makawo pachi? Ere kaka iparo ni mano | |  |  |  |
|  | timore? |  |  |  |  |
|  |  |  |  |  |
|  |  |  |  |  |  |
|  |  |  |  |  |  |
|  |  |  |  |  |  |

| 8. Be isebedo gi riekni ma ng’at ang’ata machielo ok ne nyal winjo tiende? . . . . . . | | | . . . . . . . . . . ❏ | ❏ | ❏ |
| --- | --- | --- | --- | --- | --- |
|  | ***Ka* EE:** Nyisa ewi riekni go. Ere kaka ing’eyo ni onge ng’at machielo | |  |  |  |
|  | manyalo winjo? |  |  |  |  |
|  |  |  |  |  |
|  |  |  |  |  |  |
|  |  |  |  |  |  |
|  |  |  |  |  |  |

| 9. Bende isewinjo ka gima paro oket ewiyi ma ok meki | | |  |  |  |
| --- | --- | --- | --- | --- | --- |
|  | owuon? . . .. . . . . . . . . . . . . . . . . . . . . . . . . . . . . . . . . . . . . . . . . . . . . . . . . . . . . . . . . . . . . . . . . . . . . . | | . . . . . . . . . . . ❏ | ❏ | ❏ |
|  | ***Ka* *EE***: Paro gi gin kaka mage? Ere kaka iparo ni gi | |  |  |  |
|  | donjo ewiyi? |  |  |  |  |
|  |  |  |  |  |
|  |  |  |  |  |  |
|  |  |  |  |  |  |
|  |  |  |  |  |  |

| 10. Bende iwinjo ka gima pachi okaw kod teko mamoko ma ok | | |  | |  |  |
| --- | --- | --- | --- | --- | --- | --- |
|  | Inyal chiko? . . . . . . . . . . . . . . . . . . . . . . . . . . . . . . . . . . . . . . . . . . . . . . . . . . . . . . . . . . . . . . . . . . . . . . . . . . . . . . . . | | | . . . . . . . . . . . ❏ | ❏ | ❏ |
|  | ***Ka EE:*** En ng’a kata ang’o ma chiko pachi? Ere kaka iparo ni mano | | | |  |  |
|  | timore? |  |  |  |  |  |
|  |  |  |  |  |  |
|  |  |  |  |  |  |  |
|  |  |  |  |  |  |  |
|  |  |  |  |  |  |  |

*Additional Comments or Observations:*

Psy Screen Positive if 2+ answers are shaded (OR) 3+ symptoms one time only. Do not score unless experiences described are implausible and outside of ordinary or culturally supported experiences

CDQ14

**Penjo moluwogi gin ewi kony mopogore ma dipo ni iseyudo** *(Confirm information if known)*

1. Bende isega wuoye kod jalony eweche mag ngima joma wigi olokore kaka laktar mar neko, jalony mar paro, kata jalno ma otiegi makende oyore mag konyo oganda (ng’at man kod digiri), kuom chandruok mag paro, kata kaka ne iwinjo kata kaka ne itimori?

❏ Ooyo ❏ Ee ➔ ***Ka EE:*** Ang’o mane ____________ (jalony mar ngima mar wich) no owacho? Probe for diagnosis, if any

_______________________________________________________

_______________________________________________________

_______________________________________________________

1. Bende isega wuoye gi ng’at ang’ata moro machielo ok kuom chandruok mar paro mang’eny, ligae mag ler, kata kaka ne iwinjo kata kaka ne itimori?

**❑ **Ooyo **❑ **Ee **➔Ka EE**: Ang’o mane __________ (ng’ano) owacho?

____________________________________________

____________________________________________

____________________________________________

3. Bende osega ndikni yedhe makonyo kuom chandruoge mag winjo kata paro mari kata kaka niwinjo kata kaka nitimori?

Ooyo Ee ➔ ***Ka EE:*** Yedhe mage?

 ^❏^ ^❏^ ___________________________________________________________

___________________________________________________________

4. Bende isega dhie osubtal nikech chandruok mar kaka iwinjo kata chandruok mag paro kata kaka iwinjo kata timbe gi?

❏Ooyo ❏ Ee ➔ ***Ka EE:*** *Mano ne karang’o*? *Ne orwaki e osubtal nang’o*?

____________________________________________

____________________________________________

____________________________________________

5. Bende osega thiedhi thieth moro amora ne kong’o kata yedhe mamero ji?

❏ Ooyo ❏ Ee ➔ ***Ka EE:*** Mano ne karang’o? Ne iyudo thieth mane?

_________________________________________________

_________________________________________________

6. Kuom dweche auchiel mosekalo, bende isega yudo kony moro amora mar chandruok mar kaka iwinjo kata mag paro kaka wuoyo kod jahocho kata jalony mar paro, kata muonyo yath, kata dhi osubtal ekinde machuok? ***Circle all that apply***

1. Yudo thieth kata hocho mar chandruok mar paro osubtal ti idok ot ____________________________
2. Yudo thieth mar kong’o kata yedhe ma mero ji ________________________________________________
3. Yath/yedhe mag nyaluo (ler) _____________________________________________________________
4. Rwak e osubtal ma inindo e wod

________________________________________________________________________

1. Mamoko (ler ane) _____________________________________________________________________

7. Be nitie gimoro amora maduon’g ma iparo ni onego inyisa ewi kaka iwinjo, pachi kata timbeni kuom dweche auchiel mokalo?

CDQ15

(Optional Demographic Questions)

Kwatieko, wan kod penjo mag nonro mari**.**

**1**. **Tarik mar nyuol?**_____/_____/_____

Month/Day/Year

1. **Kit chwech** *(confirm with client*)
   1. Male
   2. Female
   3. Transgender(Gath)
2. **Ne onyuoli kanye?_________________________** (*county/district)*

**4. Dhok mane ma ihero tiyogo?** *(choose one)*

1. Dho Ng’ere
2. Oswayo.
3. Dhok mar piny Kenya (ler) ____________________

4 Mamoko(ler) ________________________

**5.** En rang’iny mane mar tiegruok mane iyudo; oboke kata digiri mane mar somo ma in-go?

1. Onge
2. Primari (ok atieko)
3. Primari (Natieko)
4. Sekondari (ok atieko)

5 Sekondari (Natieko)

1. Mbalariany (Ler kit digiri: _________________________)

7 Tiegruok mar lony (ler:: ______________________)

1. Tiegruok mar tij lwedo *(ler* ________________________________)

9 Mamoko (ler:: ___________________________________)

**6. How do you identify yourself...**

1 Gay (Dichuo materore gi chuo wetene)

- Lesbian(Dhako materore gi mon wetene)
- Bisexual, attracted to both men and women(Aterora gi mon kod chuo)

4 Heterosexual, Straight(Dichuo materore gi dhako/ Dhako materore gi dichuo)

5 Not sure/ undecided/ in transition(Aonge gi adier/pok ayiero/an ediere)

6 Prefer not to say(Ayiero mondo kik awachi)

**7.** Kwan mar rangeng’ mar dendi machiegni ne en adi?

*If client gives a number write it in here* |___|___|___|

*or else use codes below*

| 1 | 0-100 | 6 | Don’t know T-cell | count but I was told it was “good” |
| --- | --- | --- | --- | --- |
| 2 | 101-200 | 7 | Don’t know T-cell | count but I was told it was “bad” 88 |
| 3 | 201-300 | 8 | Don’t know T-cell | count at all/ Don’t recall test result |
| 4 | 301-500 | 9 Client has never had T-cell CD4 test | | |
| 5 | Greater than 500 |

CDQ16

**SUMMARY SHEET (FILL OUT AFTER INTERVIEW)**

Review each section of the questionnaire and score the CDQ following the instructions in the shaded box on the bottom of the page at the end of each diagnostic module. Record all disorders for which the client scores positive on this sheet.

If the client does not score positive for any module, check here: ❏ **NO POSITIVE SCREEN IN ANY MODULE**

**DEPRESSIVE DISORDER**

❏ Positive for Major Depressive Syndrome ❏ Positive for Other Depressive Syndrome

Are client’s symptoms of depression reaction to the death of a loved one? Could symptoms be caused by medical condition, medication, or drug use? Has client ever received treatment for disorder? Other comments:

**ANXIETY DISORDER**

❏ Positive for Panic Syndrome

❏ Positive for Generalized Anxiety Syndrome

Could symptoms be caused by medical condition, medication, or drug use? Has client ever received treatment for disorder? Other comments:

**ALCOHOL ABUSE**

❏ Positive for Alcohol Abuse, past 6 months ❏ Positive for Alcohol Abuse, past 30 days

Has client ever received treatment for alcohol abuse/dependence? Has client been in a controlled environment (e.g. jail, hospital)any time during the past 6 months? in the past 30 days? Other comments:

**DRUG ABUSE**

❏ Positive for Drug Abuse, past 6 months–List drug(s) of abuse: ❏ Positive for Drug Abuse, past 30 days–List drug(s) of abuse:

Has client ever received treatment for drug abuse/dependence? Has client been in controlled environment (e.g. jail, hospital) any time during the past 6 months? In the past 30 days? Other comments:

CDQ17

**POST TRAUMATIC STRESS DISORDER**

❏ Positive on PTSD Screen

Describe traumatic events. Could symptoms be caused by medical condition, medication, or drug use? Has client ever received treatment for disorder? Other comments:

**PSYCHOSIS**

❏ Positive on Psychosis Screen

Describe symptoms. Could symptoms be caused by medical condition, medication, or drug use? Has client ever received treatment for disorder? Other comments:

**TREATMENT EXPERIENCE**

❏ Client has had professional mental health treatment or has been prescribed psych medications in the past 6 months

❏ Client is currently receiving professional mental health treatment or has been prescribed psych medications Dates of treatment? Was treatment completed? Is/was client adherent to treatment plan? Other comments:

**Interviewer Observations**

*Circle all that describe client based upon your observations during interview.*

Manifested inappropriate affect during parts of interview . . . . . . . . . . . . . . . . . . . . . . . . . . . . . . . . . . . . . . . Y . . . . . .

Unusually unkempt or bizarre in appearance . . . . . . . . . . . . . . . . . . . . . . . . . . . . . . . . . . . . . . . . . . . . . . . . . . . . . Y . . . . . .

So withdrawn into own world that s/he found it hard to answer questions . . . . . . . . . . . . . . . . . . . Y . . . . . .

Manifested unusual ways of thinking and reasoning about experiences . . . . . . . . . . . . . . . . . . . . . Y . . . . . .

Apathetic or flat in affect during interview . . . . . . . . . . . . . . . . . . . . . . . . . . . . . . . . . . . . . . . . . . . . . . . . . . . . . . . . . Y . . . . . .

Nervous and tense during interview . . . . . . . . . . . . . . . . . . . . . . . . . . . . . . . . . . . . . . . . . . . . . . . . . . . . . . . . . . . . . . . . Y . . . . . .

Intoxicated or under influence of alcohol or drugs . . . . . . . . . . . . . . . . . . . . . . . . . . . . . . . . . . . . . . . . . . . . . . . Y . . . . . .

Needle track marks . . . . . . . . . . . . . . . . . . . . . . . . . . . . . . . . . . . . . . . . . . . . . . . . . . . . . . . . . . . . . . . . . . . . . . . . . . . . . . . . . . . . Y . . . . . .

Skin abscesses, cigarette burns, or nicotine stains . . . . . . . . . . . . . . . . . . . . . . . . . . . . . . . . . . . . . . . . . . . . . . Y . . . . . .

Tremors (shaking and twitching of hands and eyelids) . . . . . . . . . . . . . . . . . . . . . . . . . . . . . . . . . . . . . . . . . . Y . . . . . .

Unclear speech: slurred, incoherent, or too rapid . . . . . . . . . . . . . . . . . . . . . . . . . . . . . . . . . . . . . . . . . . . . . . . . Y . . . . . .

Unsteady gait: staggering, off balance . . . . . . . . . . . . . . . . . . . . . . . . . . . . . . . . . . . . . . . . . . . . . . . . . . . . . . . . . . . . . Y . . . . . .

Dilated (enlarged) or constricted (pinpoint) pupils . . . . . . . . . . . . . . . . . . . . . . . . . . . . . . . . . . . . . . . . . . . . . . . Y . . . . . .

Scratching . . . . . . . . . . . . . . . . . . . . . . . . . . . . . . . . . . . . . . . . . . . . . . . . . . . . . . . . . . . . . . . . . . . . . . . . . . . . . . . . . . . . . . . . . . . . . . Y . . . . . .

Swollen hands or feet . . . . . . . . . . . . . . . . . . . . . . . . . . . . . . . . . . . . . . . . . . . . . . . . . . . . . . . . . . . . . . . . . . . . . . . . . . . . . . . . . Y . . . . . .

Smell of alcohol or marijuana on breath . . . . . . . . . . . . . . . . . . . . . . . . . . . . . . . . . . . . . . . . . . . . . . . . . . . . . . . . . . . Y . . . . .

“Nodding out” (dozing or falling asleep) . . . . . . . . . . . . . . . . . . . . . . . . . . . . . . . . . . . . . . . . . . . . . . . . . . . . . . . . . . . Y . . . . .

Agitation . . . . . . . . . . . . . . . . . . . . . . . . . . . . . . . . . . . . . . . . . . . . . . . . . . . . . . . . . . . . . . . . . . . . . . . . . . . . . . . . . . . . . . . . . . . . . . . . . Y . . . . . .

Inability to focus . . . . . . . . . . . . . . . . . . . . . . . . . . . . . . . . . . . . . . . . . . . . . . . . . . . . . . . . . . . . . . . . . . . . . . . . . . . . . . . . . . . . . . . . Y . . . . . .

CDQ18

N . . . . . . DK N . . . . . . DK N . . . . . . DK N . . . . . . DK N . . . . . . DK N . . . . . . DK N . . . . . . DK N . . . . . . DK N . . . . . . DK N . . . . . . DK N . . . . . . DK N . . . . . . DK N . . . . . . DK N . . . . . . DK N . . . . . . DK N . . . . . . DK N . . . . . . DK N . . . . . . DK N . . . . . . DK

Burns on the inside of the lips ( e.g. from smoking crack) . . . . . . . . . . . . . . . . . . . . . . . . . . . . . . . . . . . . . . Y . . . . . . N . . . . . . DK **Other comments/ observations:**

CDQ19

20
