# Supplementary material for: Adaptation of the Client Diagnostic Questionnaire for East Africa
Source: PLOS Glob Public Health. 2024 Mar 19;4(3):e0001756. doi: 10.1371/journal.pgph.0001756 (PMC10950255; doi:10.1371/journal.pgph.0001756)
Supplement: S3 Questionnaire — (PDF) [file pgph.0001756.s003.pdf]

Ebizibu by'amagara ebirengire ahari bibiri okweterana omurundi gumwe omumagara g'omuntu

Okubuuzibwa aha 'bishangirwe omu magara g'owayejumba omu mushomo.

Instructions to interviewer:

This questionnaire is designed to facilitate the recognition of the most common mental health problems found in HIV/AIDS primary care or other service settings: mood, anxiety, alcohol and drug abuse, PTSD and thought disorder.

1. Read questions as written. Additional probes may be used to ensure client understanding of the question or explore ambiguous answers.
2. For anything other than a "yes/no" answer, read the answer categories. The interviewer may need to assist the client in answering within the categories given. Never choose an answer category based on what you think the client means by their spoken response.
3. Be sure that the client is reporting symptoms experienced within the specified time period: past 4 weeks, past 6 months, or in some instances, past 30 days.
4. Within each module, proceed sequentially from question to question unless instructed either to skip to another question or to go to the next page.
5. At the end of each diagnostic module is a shaded area with instructions for scoring Positive Screen for each disorder. Scoring can be done by the interviewer or left for office use only.
6. A Summary Sheet is provided to record "positive screen" or "positive for syndrome" in the spaces provided for each diagnostic module. If no positive screen in any module, indicate in the space provided on the top of the summary sheet.
7. Space is also provided for interviewer observations and comments. Interviewer should write as detailed as possible description of positive answers to questions especially on psychosis screen. Where known, additional information that may account for symptoms (e.g. medical condition) or history of prior episodes or treatment should be indicated.
8. ***If Client indicates current suicidal feelings or becomes emotionally upset or agitated during interview, please contact a study supervisor***

RESEARCH ETHICS COMMITTEE
MBARARA UNIVERSITY OF SCIENCE AND TECHNOLOGY
SUCCEED WE MUST
P.O. BOX 1410, MBARARA

immediately.

Okwanjurira owayejumba

Ekihandiiko ky'ebibuuzo eki nikyeija kutuyamba kwetegyereza gye ebizibu ebi wakubaasa kuba oyine. -Nitubuuza ebibuuzo ebi buri omwe kugira tubaase kutunga ekishushani kirungi eky'omuringo gw'obuhwezi nari obuyambi obu twakubaasa kukuhereza. Nyaburawe gyezaho kugarukamu buri kibuuzo. Ebi oragarukyemu nibyija kubeera kyarimwe ebyekihama.

1. Ku ori kuteekateeka aha myeezi mukaaga ehweire, aho bukaba buri nk'omu kwa_______ (*reference date 6 mos prior to interview*), ebintu bibiire nibikugyendera bita abikwatireine n'aha 'mbeera eyobeire noyehurirahimu nari shi oku obeire noyehurira? Hakaba hariho obunaku obuwabeire nohurira oyine obusaasi nari enaku erengyesereize? Kandi shi obweire bwoona obu washangire otiinire, oyine ebirikukutiinisa, nari oyeraarikiriire ahabintu? Hariho obweire obuwabeire oyine amaani mingyi otarikushutama hamwe nari oyine amashemererwa mingyi kugira ngu okaba otakubaasa kukyendeezaho?

2. Haruho ekintu kyoona ekyakubeireho omubweire obwo ebyabeire byeine akakwaate n'okuwabeire noyehurira (ebiwabeire nokora) nka (okuguubwa kubi, okuba otiinire nari oyerarikiriire, okuba oyine amashemererwa mingyi, ebindi... refer to symptoms)? Ekintu kyoona ekyabeire okukira munonga ekikugumiire nari kirikukwerarikiriza munonga?

3. Omu myeezi mukaaga ehweire heine omuntu weena owu ogambireho nawe ahabikwatireine nebizibu byaawe by'okukwatwaho, okutiina kwaawe, nari omuringo ogu wabeire noyehuriramu, nari ebiwabeire nokora? Ku eraabe eri eego, okagambaho nooha? (Probe) Heine omuntu weena omukugu owu wagambireho nawe nk'omushaho nari omuhumuriza? Bakakigambahoki?

RESEARCH ETHICS COMMITTEE
MBARARA UNIVERSITY OF SCIENCE AND TECHNOLOGY
SUCCEED WE MUST
P.O. BOX 1410, MBARARA

Interviewer: *If client describes symptoms or treatment history, let him/her know that you will be talking about this in more detail later in the interview. All screening and appropriate symptom questions must be asked even though topic was discussed in overview. Confirm answers already known.*

Hati ebibuuzo bimwe ebikwatireine n'embeera zaawe eziwabeire noyehuriramu nari shi okuwabeire noyehurira. Omukwezi okuhweire (esande 4 ezihweire) hakaba haruho obweire bwoona obu .....

	Ngaaha, tiburiho nakakye	Amazooba mingyi	kurengamu	Heihi buri eizooba
1. Obu wabeire nohurira ogweirwe kubi, oteine 'maani nari ori ahansi, oyine enaku erengyesereize, nari kuba oyehweire amatsiko. **KU ERAABE ERI EEGO,** N'emirundi engahi obuwahureire otyo?	☐	☐	☐	☐
2. Obu wabeire oyine okushemezibwa nari okushemererwa kukye omu kukora ebintu? ***KU ERAABE EEGO,*** n'emirundi engahi obu wahureire otyo?	☐	☐	☐	☐

If client answers "No, Not at all" to both questions, go to next page.

3. Ni ryaari obu otandika kwehurira oti (omu biro ebi ebihweire)?

4. Kikamara obweire burikwingana ki _______ kikamara nk'esande ibiri? ☐ Eego ☐ Ngaaha

Omubweire obwo, nemirundi engahi obu watungiremu okuteganisibwa ahabwa:

	Ngaaha, tiburiho nakakye	Amazooba mingyi	kurengamu	Heihi buri eizooba
5. Okutunga obuzibu omu kubyama nari kuremwa kuguma ogwejegyeire? Nari kugwejegyera munonga?	☐	☐	☐	☐
6. Okuhurira oruhire ninga kuba oyine amaani makye?	☐	☐	☐	☐

RESEARCH ETHICS COMMITTEE
MBARARA UNIVERSITY OF SCIENCE AND TECHNOLOGY
SUCCEED WE MUST
P.O. BOX 1410, MBARARA

7. Okuhurira otakwenda kurya? Nari okurya munonga?

8. Okwehuriraho kubi– Nari okwereeba nk'owaremirwe nari okeereebya nari okareebya abeeka yaawe.

9. Okugumirwa omu kuta omutima aha'bintu, nk'okushoma ihurire, okureeba za tiivi, omuntu ariyo nahereza eby'okugyenderaho nari okukuratira ekigaaniro?

10. Okugyenda nari kugamba mpora kugira ngu n'abantu abandi nibabaasa kuba baakimanyire? Nari ekishungaine n'ekyo– okukangaza nari obutagumisiriza kugira ngu okaba noyetoroora munonga ekitari kya bureijo?

11. Ebiteekateeko by'okugira ngu okabeire origye waaba ofiire nari ebiteekateeko by'okwehitsyaho obuhurizi omu muringo gwona.?

Maj Dep Syn if 2 weeks (Q4) is "yes" (AND) answer to question 1 or 2 is shaded (AND) 5+ of answers to any of Q. 1, 2, 5 - 11 are shaded; Other Dep Syn same but only 2+ of the answers to Q. 1, 2, 5 - 11 are shaded.

Hati ebibuzo bimwe ebikwatireine n'okutiina...

	EEGO	NGAAHA
1. Omu sande 4 ezihweire, obeire wakwatsirwe okutiina - okw'ahonaaho, okuhurira obwooba nari okujabiirira otiinire?		

If client answers "NO" go to next page.

2. Eki kikaba kyaraabireho enyimaho?		
3. Okukwatwa oku heine obumwe obu kuri kweija ahonaaho ekitarariireine— nk'omubweire obu orikuba otakuteekateeka kugira okutiina nari obutatebekana?		
4. Okukwatwa oku heine obu kuri kukuteganisa munonga? Nohurira oyerarikiriire okugarukamu kukwatwa ogundi?		

Teekateeka ahakukwatwa kwawe okubi buzima okuhererukire

5. Okaburwa omwitsyo?		
6. Omutima gwawe gukaba nigwiruka, nigushekura, nari nigugurukyeera?		
7. Okaba noshaasha ekifuba nari kuhurira omigirwe (puresha)?		
8. Okatuutuka?		
9. Okaba nohurira oine nk'ekyakuniga?		
10. Okaba ohurira wagira okwerabwa kw'omuriro nari okushushwa ?		

RESEARCH ETHICS COMMITTEE
MBARARA UNIVERSITY OF SCIENCE AND TECHNOLOGY
SUCCEED WE MUST
P.O. BOX 1410, MBARARA

11. Okagira orusheshemi nari okuteganitsibwa omunda, nari kuhurira nk'orikuza kugira ekiirukano? ☐ ☐
12. Okahurira oruzengyerera, orink'orikwenda kugwa, nari okagwa? ☐ ☐
13. Okagira nk'obuntu burikukucumacumita omubiri (nk'obuhitirizo na za piini) nari okushanyarara omu bicweeka by'omubiri gwaawe? ☐ ☐
14. Okaba nokankana nari orikutetema? ☐ ☐
15. Okaba otiinire ngu waaza kufa? ☐ ☐

Pan Syn if answers to Q. 1,2,3 and 4 are 'Yes' (AND) 4+ symptoms during an attack (Q. 5-15)

Okurenga omu sande 4 ezihweire, n'emirundi engahi obuwateganitsibwe ahabwa:

	Ngaaha, tiburiho nakakye	Amazooba mingyi	kurengamu	Heihi buri eizooba
1. Okuhurira otiinire, oyerarikiriire, ori aharusinga/rubaju oyemereize omutima munonga ahabintu bitari kushushana?	☐	☐	☐	☐

If client answers "Not at all" go to next page.

	Ngaaha, tiburiho nakakye	Amazooba mingyi	kurengamu	Heihi buri eizooba
2. Okuhurira nokangaza kugira ngu okushutama hamwe nikiguma?	☐	☐	☐	☐
3. Okuruha juba?	☐	☐	☐	☐
3. Enyama zomubiri okwerarikirira, okuhururwa, nari oburonda?	☐	☐	☐	☐
5. Okutunga obuzibu omu kubyama nari kuremwa kuguma ogwejegyeire?	☐	☐	☐	☐
6. Okugumirwa omu kuta omutima aha'bintu, nk'okushoma ihurire, okureeba tiivi, omuntu ariyo nahereza eby'okugyenderaho nari okukuratira ekigaaniro?	☐	☐	☐	☐
7. Okurahuka kugubwa kubi nari ekiniga kya heihi?	☐	☐	☐	☐

Other Anx Syn if answer to Q. 1 is shaded (AND) 3+ answers to Q. 2-7 are shaded

RESEARCH ETHICS COMMITTEE
MBARARA UNIVERSITY OF SCIENCE AND TECHNOLOGY
SUCCEED WE MUST
P.O. BOX 1410, MBARARA

Ebyakurataho ni bimwe ahabibuuzo ebikwatireine n'okunywa amaarwa hamwe n'enkozesa y'ebindi bintu. Nitubuuza ebibuuzo ebi nk'ekicweeka ekiri kugamba aha 'magara ga buri omwe. Buri kimwe eki orangambire nikiragiirwa kuba kiri ekihama kandi kirinzirwe.

1. Omu myeezi mukaaga ehweire, n'emirundi engahi ey'orikunywamu biya, Vinyo nari ebindi by'okunywa ebitabwiire?

Tikikabahoga	Ahansi y'okwezi kumwe	Buri kwezi	Buri sande	Emirundi 3 omu sande	Buriizooba
☐	☐	☐	☐	☐	☐

If client <u>never</u> drinks alcohol, go to last alcohol question - Q.13 next page.

*****Please use the worksheet "Calculation of Number of Drinks Consumed" for Q.2*****

2. Nokira kunywa ebyokunywa bingahi aha 'mazooba ago obu ori kunywa?

Kimwe	Bibiri	Bishatu	Bina	Bitaano	Kurenga omuri bitaano
☐	☐	☐	☐	☐	☐

Heine ekintu kyoona ekikubeireho omu bintu ebyaaza kugambwaho <u>kurenzya omurundi gumwe omu myeezi 6 ehweire</u>, obwo ni kweiha (______________) okuhitsya eriizooba?

	**EEGO**	**NGAAHA**
3. Okanywa amaarwa nobu omushaho araabe yabeire ahabweire ngu orekyeraaho kunywa ahabw'ekizibu ekyamagara gaawe geine?	☐	☐
4. Okanywa amaarwa, gakusiinza, okaraara enkyeera ahabw'amaarwa obwo ori ahamurimo, orikuza aheishomero, nari orikureeberera abaana nari endeijo myoga?	☐	☐
5. Okafeerwa nari okakyererwa aha'kintu kikuru ahabw'okuba okaba nonywa nari oreire enkyeera ahabw'amaarwa.	☐	☐
6. Okaba oyine ekizibu ky'obutakwatagana n'abandi mwaaba nimunywa amaarwa?	☐	☐
7. Okavugaho ekiiruka (akamotoka akakye, ekihango, nari pikipiki) nari okakoraho n'ebyooma ebirikuremeera waheza kutunga ebyokunywa byingyi nari bwanyima yokunywa munonga?	☐	☐

Alc Abu if 1+ answers to Q. 3-7 are Yes (OR) 5+ drinks a day weekly or more often

Omu BIRO 30 EBIHWEIRE, obwo ni, kweiha obweire obu omukwa... (____________________)...

8. Ni amazooba angahi obu onyweireho ekyokunywa kirimu amaarwa? ____________

If client never drank alcohol in the past 30 days, go to last alcohol question –Q. 13 below

Omu biro 30 ebihweire...

	EEGO	NGAAHA
9. Ogiziremu ekiteekateeko ky'okugira ngu oshemereire kukyendeeza aha minyweere yaawe y'amaarwa?	☐	☐
10. Heine omuntu weena owayetomboitsire ahabw'okunywa kwaawe?	☐	☐
11. Heine obu wahuriire noyecweera omushango nari okagira ekiniga ahabw'okunywa kwaawe?	☐	☐
12. Haruho eizooba ryoona eri wanyweireho ebyokunywa bitaano nari birikurengamu bya biya, vinyo, nari ebindi byokunywa ebitabwiire?	☐	☐
**ASK EVERYONE**		
13. Haruho obu eiwe nari ondiijo 'muntu akukwatsireho munonga yarateekateekire ngu oyine ekizibu namaarwa?	☐	☐

Alc Abu 30 day if 2+ answers to questions 9-12 are YES

RESEARCH ETHICS COMMITTEE
MBARARA UNIVERSITY OF SCIENCE AND TECHNOLOGY
SUCCEED WE MUST
P.O. BOX 1410, MBARARA

Hati aha haruho ebibuuzo bimwe ebikwatsire aha nkozesa y'ebikutokooza 'bwongo. (*Remind client of confidentiality*)

Oyijukye ngu buri kimwe eki orangambire nikiragiirwa kuba kiri ekihama kandi kirinzirwe..

Waarakozeiseho ebitokooza 'bwongo byoona ebyaaza kugambwaho, nobu gwakuba omurundi gumwe?

	Warakozeiseho		Ku eraaabe eri EEGO ahabwa buri kitokooza bwongo buuza: Omu MYEEZI 6 EHWEIRE, n'emirundi engahi obu okozeiseho (ekitokooza 'bwongo)?					
	**Eego**	**Ngaaha**	**Tikikabahoga**	**Ahansi y'okwe ezi kumwe**	**Buri kweezi**	**Buri sande**	**Emirundi 3 omu sande**	**Buriizooba**
1. Enjaayi (hashish, cannabis, weed, bhang, bangi, ganja, sensi, boza, ikhendi)								
2. Emibazi erikukyendeeza obusaasi nari eyibakurya kishuma ekareeta amashemererwa								
3. Emibazi eyibakureesa ekasinza								
4. Heroin, brown, sugar, white cap, white crest, unga								
5. Emibazi erikuhindura enteekateeka nari emitwarize y'omuntu otatungire 'ndagiriro y'omushaho, nari erikurenga ahayomushaho eyi yakugambeire ...								
6. Emibazi erikugwejegyeza (valium, stilnox, cough syrup) eteine 'ndagiriro nari erikurenga ahayomushaho yakugambeire....								
7. Emibazi erikuretera enkora y'omubiri yayeyongyera(metham								

RESEARCH ETHICS COMMITTEE
MBARARA UNIVERSITY OF SCIENCE AND TECHNOLOGY
WE MUST SUCCEED
P. O. BOX 1410, MBARARA

phetamine, uppers, speed, ice) eteine 'ndagiriro nari erikurenga ahayomushaho yakugambeire ….								
8. Emibazi erikureetera ameisho gareeba ekintu encure (PCP, angel dust, ecstasy, mushrooms, LSD)								
9. Emibazi eyi barikwitsyamu akasinza (sprays, glue)								
10. Ebindi (see Appendix 2)								

KU ORAABE WAARAKOZEISEHO EBITOKOOZA 'BWONGO BYOONA: **EEGO** **NGAAHA**

11. Waaratungireho ekitokooza 'bwongo kyoona ekyateirwe omu omumubiri gwaawe nk'ekikatu, nari ogubatsindikire ahansi y'oruhu rwaawe rwomubiri barikwejunisa empitirizo,nobu gwaakuba omurundi gumwe? ☐ ☐

KU ORAABE WARAKOZEISEHO EMPITIRIZO:

12. Waaratungireho omubazi gwoona ogw'ekitokooza 'bwongo ogwateirwe omu omumubiri gwaawe nk'ekikatu, nari ogubatsindikire ahansi y'oruhu rwaawe rwomubiri barikwejunisa empitirizo eshaha yoona omu myeezi 6 ehweire? ☐ ☐
    Ku eraabe eri eego, mubazi ki?______________________________ ☐ ☐

If No Drug Use in 6 MONTHS go to page 11 TRAUMA

Buuza abarweire boona abakozeiseho ebitokooza 'bwongo byoona omu myeezi 6 ehweire

Omu 'bintu ebyaaza kugambwaho heine ekyaraakubeireho kyoona kurenzya omurundi gumwe omu myeezi mukaaga ehweire, obwo ni kuruga (_____________________) kuhitsya eriizooba?

	EEGO	NGAAHA
13. Okakozesa ebitokooza 'bwongo nobu omushaho araabe yahabwiire ngu orekyeraaho okubikozasa ahabw'ekizibu eki oyine omumagara gaawe?	☐	☐
14. Okakozesa ebitokooza 'bwongo, wasinda, waraara enkyeera ahabw'ebitokooza 'bwongo obwe orihamurimo, ori kuza aheishomero, orikureberera abaana nari endiijo myooga?	☐	☐
15. Okafeerwa nari okakyerererwa aha'bintu bikuru ahabwokuba okaba okozeise ebitokooza 'bwongo nari okaba oreire enkyeera ahabw'ebitokooza 'bwongo.?	☐	☐
16. Okaba oyine ekizibu ky'okuguma omu 'bandi bantu obu mwabeire nimukozesa ebitokooza 'bwongo?	☐	☐
17. Okavuga motoka waheza kukozesa ebitokooza 'bwongo	☐	☐
18. Okagira ebizibu by'amateeka ahabw'okukozesa ebitokooza 'bwongo (Poliisi okukutaahataahamu, kutwaarwa omu kooti nari heine owa kutabareize, ebizibu byoona ebyeine akakwaate n'ekiragiro.)	☐	☐

DRUG ABU if 1+ answers to Q 12 - Q 18 are Yes (OR) Heroin, Coke/Crack or Methamphetamine 3+ per week

Omu BIRO 30 EBIHWEIRE, obwo ni, kweiha obweire obu omu kwa (_______________)

____

Ni amazooba angahi obu okozeise

14. Enjaayi ☐

15. Emibazi erikukyendeesa obusaasi nari eyibakurya ekareeta kishuma amashemererwa ☐

☐

RESEARCH ETHICS COMMITTEE
MBARARA UNIVERSITY OF SCIENCE AND TECHNOLOGY
SUCCEED WE MUST
P.O. BOX 1410, MBARARA

16. Emibazi eyibarikureesa ekabasinza

17. Heroin or speedball ☐

18. Emibazi erikugwejegyeza ☐

19. Emibazi erkuretera enkora y'omubiri yayeyongyera ☐

20. Emibazi erikuretera ameisho gareeba ebintu encure ☐

21. Emibazi eyi barikweitsyamu ekasinza ☐

If client <u>never</u> used any drug in past 30 days, go to next page

---

Omu biro 30 ebihweire....

	EEGO	NGAAHA
22. Heine obu wateekateekire ngu oshemereire kukyendeeza aha 'nkozesa yaawe Y'ebitokooza 'bwongo?	☐	☐
23. Heine omuntu weena owayetomboitsire aha 'nkozesa yaawe y'ebitokooza 'bwongo?	☐	☐
24. Wahureire noyecweera omushango nari okagira ekiniga ahabw'enkozesa yaawe y'ebitokooza 'bwongo?	☐	☐
25. Wakozeise ebitokooza 'bwongo byoona emirundi 3 nokukiraho omu sande nari kukiraho emirundi myingyi?	☐	☐

ASK EVERYONE

Hati ebibuuzo bimwe ebikwatireine n'aha bintu bibi munonga nari ebirikutiinisa ebyakubaasa kuba byaakubeireho.

Abantu keingyi barabire omu 'bintu birikureeta ekiroonda omu mutwe. Nimanyisa bibi munonga, birikutiinisa. Ninza kushoma orukarara rw'ebintu ebyakubaasa kuba byabeire ah'abantu obumwe nobumwe. Nyaburawe ngambira kuwakubaasa kuba waraabirabiremu....

	EEGO	NGAAHA
1. Butandu egumire (omuka, n'emotoka, na pikipiki) nari omuriro omuka nari ahamurimo gwaawe . . .	☐	☐
4. Eby'obuhangwa ebirikushisha ensi nka emiyaga yamaani, omutsitsa gw'amaani, eihunga ribi, omwegyemure nari ebindi nk'ebyo ebirikushisha ensi....	☐	☐
5. Okwejumbira omukurwaana omu rutaro	☐	☐
6. Omukundwa waawe kukuteera nari kukurwanisa nari kukutwariza kubi ahamagara gaawe ga bukuru	☐	☐

RESEARCH ETHICS COMMITTEE
MBARARA UNIVERSITY OF SCIENCE AND TECHNOLOGY
SUCCEED WE MUST
P.O. BOX 1410, MBARARA

7. Ondiijo 'muntu atari mukundwa waawe kukuteera, kukutwariza kubi nari kukwiiba omu magara gaawe ga bukuru. ☐ ☐

8. Okuteerwa nari kukutwariza kubi nk'omwaana . . . ☐ ☐

9. Okureeba abantu nibateera abandi nari nibabagweisa kubi omuka yanyu obuwabeire nookura... ☐ ☐

10. Okukuteera barikwenda kukuta omu by'omubonano nari okuhamba omu magara gaawe ga bukuru . . . ☐ ☐

11. Okukuteera barikwenda kukuta omu by'omubonano nari okuhamba nk'omwaana . . . ☐ ☐

12. Okureeba omuntu arikuteerwa nari arikubonabonetsibwa... ☐ ☐

13. Okureeba omuntu ayine obuhuta bw'amaani nari kubonabonetsibwa bari kumwiita . . . ☐ ☐

14. Okufeerwa omwaana ahabwa rufu . . . ☐ ☐

15. Okurugwamu enda ☐ ☐

16. Ekindi kintu kibi munonga nari ekirikutiinisa ekyakubaasa kuba kyakubeireho. Kigambeho ☐ ☐

If client answers "NO" to all questions go to Page 13 PSY

If client answers "YES" to one or more questions go to the NEXT PAGE

If client answers "YES" to <u>ONLY ONE</u> event listed on the previous page, ask Q. 1A

1A.Wangambira ahabwiire obu wa ______________________________(Kigambeho ekyakubeireho).
Ninyenda kukubuuzaho kakye ogundi ahakyakubeireho . . . . **skip to Q.2**

If client answers "YES" to <u>MORE THAN ONE</u> event listed on the previous page, ask Q. 1B

1B.Wangambira omuhendo gw'ebintu byingyi ebyakubeireho. Omuri ebi ebyakubeireho, nikyi ekyakirizeyo kuba kibi munonga nari kutiinisa ahabwaawe? ____________________________ *(Gamba ahabyabeireho nari ebyeine akakwaate n'okukuratana kw'ebyabeireho ebi owayejumba omu mushomo aragambeho)*

Ninyenda kukubuuzaho kakye ogundi ahabikwatireine n'ebyabeireho ebi (okukuratana kw'ebyabeireho)...

2. Okatiina ota...

Tikiraabeireho	Kakye mbwenu	Kubi	Kubi munonga	Nkatiina kakye nfe
☐	☐	☐	☐	☐

Omu myeezi mukaaga ehweire...

RESEARCH ETHICS COMMITTEE
MBARARA UNIVERSITY OF SCIENCE AND TECHNOLOGY
SUCCEED WE MUST
P. O. BOX 1410, MBARARA

	EEGO	NGAAHA
3. Noguma nokyijuka nobu orikuba otarikwenda kwijuka?	☐	☐
4. Notunga ebirooto bibi ahabw'ekyo?	☐	☐
5. Ebintu ebirikukikweijutsya nibikugweisa kubi nari oteganitsibwa?	☐	☐
5. Waaratungireho okwerabwa – okuba oryaaho omukaanyabwaanya okashusha oti ekyabeireho obwo kiriyo nikigarukamu ogundi?	☐	☐
6. Noyemereza omutima munonga kugira ngu nikibaasa kugaruka kikabaho?	☐	☐
7. Noyehara ebintu ebirikukikweijutsya?	☐	☐
8. Nokira obumwe nobumwe kugira okugumirwa omukweijuka kyonyini ekyabeireho?	☐	☐
9. Nohurira ori wenka nobu orikuba oyine abandi bantu, nari ohurira otaanisibwe kuruga omu 'bandi bantu?	☐	☐
10. Noshanyarara nari ohurira oteine kintu kyoona oki orikubaasa kuhurira nka kyakukwataho?	☐	☐
11. Noguraaguruka, otiina orahuka kwerarikirira nari oguma ori nk'oyine ekyorinzire nobu kyaakuba kitarikwetaagisa?	☐	☐

******If client answers "YES" to MORE THAN ONE event on the previous page, ask Q.1C in Appendix 3 ******

PTS Syn if answer to 2 is "Bad" or worse (AND) 1+ answers to Q 3-6 (AND) 2+ answers to Q.8-11 are YES

Hati ninza kukubuuza ahanyikiriza ninga enteekateeka eyi abantu bamwe beine. Abantu abamwe beine enteekateeka egi nari enyikiriza egi baaba baherize kunywa ahamaarwa nari kukozesa ebitokooza 'bwongo. Ninyenda kumanya yaaba waaragizireho enteekateeka egi nari enyikiriza egi omu SANDE 4 EZIHWEIRE (ebiro 30) obu OBEIRE OTA kunywa 'maarwa nari kukozesa ebitokooza bwongo.

Omu sande 4 ezihweire, n'emirundi engahi . . .

	Tikikabahoga	Omurundi gumwe	Kurenga omurundi gumwe
1. Wahuriireho orwaari nari amaraka agu abantu abandi barikugira ngu bo tibarikuhurira? **KU ERAABE ERI EEGO,** ngambira kikaba kiri ki ekiwahureire? Kuriraabe ryabeire riri eiraka: ri/gakagamba ki? Ri/gakakugambira kugira ekiwakora? Kukoraki ? Eiraka eri nirishusha nkerikuba riri omu mutwe gwaawe nari niriruga aheeru?	☐	☐	☐

RESEARCH ETHICS COMMITTEE
MBARARA UNIVERSITY OF SCIENCE AND TECHNOLOGY
SUCCEED WE MUST
P.O. BOX 1410, MBARARA

2. Heine obu wateekateekire ngu haruho abantu ababeire nibenda kukuhutaaza nari kukugweisa kubi? **KU ERAABE ER EEGO**, Bakaba bari bantu ki? Ahabwenki nibeenda kukugweisa kubi? Obwooba bwaawe ahabw'eki niburetera wagumirwa kuruga omuka yaawe nari ahu orikukira kuraara? ☐ ☐ ☐

3. Heine obu wateekateekire ngu haruho ekintu ekiriyo nikikubaho ekitari kya butoosha nari ebintu ebikwetoreire biriyo nibihinduka? **KU ERAABE ERI EEGO**, Nobaasa kugira ekiwangambira ekikikwatsireho? Noteekateeka ngu abaantu bakunaamiire? Noreeba ebintu bikweiniire amakuru gatari ga butoosha? Nk'enamba nari obumanyitso 'bwahanguuto nari ebindi nk'ebyo? ☐ ☐ ☐

4. Heine obuwatungire okworekwa ninga okareeba ebintu ebi abandi 'bantu barikugira ngu tibari kubireeba? **KU ERAABE ERI EEGO**, Ngambira ahaky'orebire. Eki nikibaho waaba osiimukire? Nikibaho nkahi? Nooreeba omuntu oherize kufa ? ☐ ☐ ☐

5. Heine obuwahureire ngu oyine amaani agatari ga 'butosha agu abandi 'bantu bateine? **Ku eraabe eri EEGO**: Ngambira aha 'maani aga. Nigataana gata nebi abandi bantu barikubaasa kukora ? Amaani aga ogakozeise ota? ☐ ☐ ☐

6. Heine obu wateekatekire ngu otungire omuzimu nari sitaane?. **KU ERAABE ERI EEGO**, Nobaasa kungambira ahari ekyo? heine ekintu kyoona eki omuzimu/sitaane bikureteire wakora? Nikintuki? ☐ ☐ ☐

RESEARCH ETHICS COMMITTEE
MBARARA UNIVERSITY OF SCIENCE AND TECHNOLOGY
SUCCEED WE MUST
P.O. BOX 1410, MBARARA

Omu sande 4 ezihweire, n'emirundi engahi . . .

	**Tikikabahoga**	**Omurundi gumwe**	**Kurenga omurundi gumwe**
7. Heine obu ohuriire ebiteekateeko byaawe byatwaarwa amaani agarikuruga aheeru nari agatari omureiwe? **KU ERAABE ERI EEGO**, Nooha nari niki ekirikutwaara ebiteekateeko byaawe? Nogira ngu ekyo nikibaho kita?	☐	☐	☐
8. Waagizire ekirooto nari ekiteekateeko eki hateine omuntu weena ondiijo orikubaasa kukyetegyereza? **KU ERAABE ERI EEGO**, Ngambira ahabiteekateeko ebi. Noteekateeka ota ngu tiheine muntu weena ondiijo orikubaasa kukikyenga?	☐	☐	☐
9. Waagizire okuteekateeka ngu ebiteekateeko bikateibwa omu mutwe gwaawe ebyabeire bitari byaawe ahabwaawe? **KU ERAABE ERI EEGO**, Nibeiha bimwe ahabiteekateeko ebi? Nogira ngu ebiteekateeko ebi nibihika bita omu mutwe gwaawe?	☐	☐	☐
10. Heine obu ori kuteekateeka ngu okwetegyereza kwaawe kukatwaarwa amaani ogu otarikubaasa kukwataho? **KU ERAABE ERI EEGO**, Nooha nari niki ekirikuvuga okwetegyereza kwaawe? Nogira ngu eki nikibaho kita?	☐	☐	☐

Additional comments or observations......

RESEARCH ETHICS COMMITTEE
MBARARA UNIVERSITY OF SCIENCE AND TECHNOLOGY
SUCCEED WE MUST
P.O. BOX 1410, MBARARA

Psy Screen Positive if 2+ answers are shaded (OR) 3+ symptoms one time only. Do not score unless experiences described are implausible and outside of ordinary or culturally supported experiences

Ebi ebibuuzo ebyakurataho nibikwaata aha 'buhereza butarikushushana obu wakubaasa kuba watungire*(hamya amakuru waaba nogamanya.)*

1. Waaragambireho nomukugu w'ebyamabagara g'eby'omu 'mutwe nka omushaho omukugu ahabw'endwaara z'omutwe, Omukugu ahabw'eby'okuteekateeka nari kugambira aha mutima, nari omukugu otendekirwe kukora omu 'bantu (omuntu oyine diguri), ahabikwatireine n'ebizibu by'okukwatwaho, Okutiina kwaawe, nari omuringo ogu wabeire noyehuriramu nari okuwabeire notwaaza?

EEGO NGAAHA **KU ERAABE ERI EEGO**, Omukugu w'ebyagara g'omu mutwe ________________akagambaki?

Probe for diagnosis, if any

☐ ☐

2. Waaragambireho n'omuntu weena owu tutaagambaho omu kibuuzo ekyaharuguru ahabikwatireine nebizubu by'okukwatwaho, okutiina kwaawe, nari omuringo ogu wabeire noyehuriramu nari okuwabeire notwaaza?

EEGO NGAAHA **KU ERAABE ER EEGO**, Ogwo 'muntu____________akagambaki?

☐ ☐

3. Barakuragiriireho emibazi yokukuyamba ahabw'ebizibu byaawe by'okukwatwaho nari ebizibu by'ebitekateeko nari okuwabeire noyehurira nari noyetwaazawa?

EEGO NGAAHA **KU ERAABE ERI EEGO**, ni mibazi/mubazi ki?

☐ ☐

4. Waaragiireho omweirwariro ahabw'ebizibu by'okukwatwaho nari ebizibu by'ebiteekateeko nari emiringo eyi wabeire noyehuriramu nari oku wabeire notwaaza?

EEGO NGAAHA **KU ERAABE ERI EEGO**, Ni ryaari? Ahabwenki bakutweire omw'eirwariro?

☐ ☐

RESEARCH ETHICS COMMITTEE
UNIVERSITY OF SCIENCE AND TECHNOLOGY
SUCCEED WE MUST
MBARARA
P.O. BOX 1410, MBARARA

5. Waaratungireho ekika kyoona ky'obujanjabi ahabw'ekizibu ky'amaarwa nari ekitokooza 'bwongo?

EEGO **NGAAHA** **KU ERAABE ERI EEGO**, Ni ryaari? Ni kika ki ky'obujanjabi ekiwatungire?

☐ ☐

6. Omu <u>myeezi 6 ehweire</u> watungire obuyambi bwoona ahabw'okukwatwaho nari okugumirwa omu 'biteekateeko nk'okugambaho n'omukugu w'ebyokuteekateeka nari omushaho wabarweire b'emitwe nari okumira emibazi nari okuza omweirwariro okamarayo akeire?
Circle all that apply

a. Nkatunga okuragurirwa nataaha nari nahumurizibwa ahabwebizibu by'ebiteekateeko____________________

b. Nkatunga okuragurirwa ahabw'amaarwa nari ekitokooza 'bwongo

____

c. Emibazi/ebishaka (Bigambeho)

____

d. Nkatunga ekitanda omweirwariro

____

Ekindi (Kigambeho)

____

7. Haruho ekintu kyoona ekikuru eki orikuteekateeka ngu oshemereire kuba nongambiraho ekikwatereine nembeera ez'orikwehuriramu, oku orikwehurira, eki orikuteekateeka nari emiringo eyi orikwetwaazamu omu myeezi 6 ehweire?

(Optional Demographic Questions)

Ahamuheru, tweineyo ebibuuzo bikye ebirikugamba ahabirikukukwatahoFinally.

1. Okazaarwa ebiro bingahi?_____/_____/_____ Omweezi/Eizooba/Omwaaka

2. Obuhangwa*(Hamya n'omurweire*)

a. Omusheija
b. Omukazi
c. Nabuzabuza timukazi timusheija

3. Okazarirwa nkahi?__________________________ (*Ishaza/disiturikiti)*

4. Nokunda kugamba rurimi ki? *(Tooranamu kimwe)*

a. Orujungu
b. Oruswahiri
c. Kenyan(Rugambeho)
d. Orundi (Rugambeho)

5. Omweishomero okahika omukyakangahi? Diguri ninga dipuroma eyaheiguru eyiwatungire neeha, kuyakuba eriho?

a. Tindashomire
b. Purimare(ekicweeka kyaayo)
c. Purimare (nkagiheza)
d. Siniya (ekicweeka kyaayo)

RESEARCH ETHICS COMMITTEE
MBARARA UNIVERSITY OF SCIENCE AND TECHNOLOGY
SUCCEED WE MUST
P. O. BOX 1410, MBARARA

d. Siniya(nkagiheza)
e. Yunivasite (Gamba ahari diguri)
f. Emyooga eyokutendekwa (Gigambeho)
g. Nkashoma eby'okwekozesa *(Bigambeho)*
h. Ebindi (Bigambeho)

6. Iwe noyemanyira nkahi...

a. Omusheija w'ekitingwa
b. Omukazi w'ekitingwa
c. Nimbuzabuza, ninkundwa boona abasheija nabakazi
d. Ninkundwa
e. Tindikumanya/Tinkashaziremu/ Ndimu nimpinduka
f. Tindikwenda kukigamba

7. Omubaro gw'abaserukare baawe nari obutafaari oguhererukikire niguuha?

If client gives a number write it in here |___|___|___| or else use codes below

a. 0-100
b. 101-200
c. 201-300
d. 301-5001
e. Aheiguru ya 500
f. Tindikumanya omubaro gw'abaserukare kwonka bakangira ngu gukaba guri "murungi"
g. Tindikumanya omubaro gw'abaserukare kwonka bakangira ngu gukaba guri "mubi"
h. Tindikumanya mubaro gw'abaserukare nakakye/Tindikwijuka ebyarugire omukukyebera
i. Omurweire takakyezahoga abaserukare/obutafaari

RESEARCH ETHICS COMMITTEE
MBARARA UNIVERSITY OF SCIENCE AND TECHNOLOGY
SUCCEED WE MUST
P. O. BOX 1410, MBARARA

SUMMARY SHEET (FILL OUT AFTER INTERVIEW)

Review each section of the questionnaire and score the CDQ following the instructions in the shaded box on the bottom of the page at the end of each diagnostic module. Record all disorders for which the client scores positive on this sheet.

If the client does not score positive for any module, check here:

❑ **NO POSITIVE SCREEN IN ANY MODULE**

DEPRESSIVE DISORDER

❑ Positive for Major Depressive Syndrome

❑ Positive for Other Depressive Syndrome

Are client's symptoms of depression reaction to the death of a loved one? Could symptoms be caused by medical condition, medication, or drug use? Has client ever received treatment for disorder? Other comments:

ANXIETY DISORDER

❑ Positive for Panic Syndrome

❑ Positive for Generalized Anxiety Syndrome

Could symptoms be caused by medical condition, medication, or drug use? Has client ever received treatment for disorder? Other comments:

ALCOHOL ABUSE

❑ Positive for Alcohol Abuse, past 6 months

❑ Positive for Alcohol Abuse, past 30 days

Has client ever received treatment for alcohol abuse/dependence? Has client been in a controlled environment (e.g. jail, hospital)any time during the past 6 months? In the past 30 days? Other comments:

RESEARCH ETHICS COMMITTEE
MBARARA UNIVERSITY OF SCIENCE AND TECHNOLOGY
SUCCEED WE MUST
P.O. BOX 1410, MBARARA

DRUG ABUSE

☐ Positive for Drug Abuse, past 6 months–List drug(s) of abuse: ______________________________

☐ Positive for Drug Abuse, past 30 days–List drug(s) of abuse: ______________________________

Has client ever received treatment for drug abuse/dependence? Has client been in controlled environment (e.g. jail, hospital) any time during the past 6 months? In the past 30 days? Other comments:

POST TRAUMATIC STRESS DISORDER,

☐ Positive on PTSD Screen

Describe traumatic events. Could symptoms be caused by medical condition, medication, or drug use? Has client ever received treatment for disorder? Other comments:

PSYCHOSIS

☐ Positive on Psychosis Screen

Describe symptoms. Could symptoms be caused by medical condition, medication, or drug use? Has client ever received treatment for disorder? Other comments:

TREATMENT EXPERIENCE

☐ Client has had professional mental health treatment or has been prescribed psych medications in the past 6 months

☐ Client is <u>currently</u> receiving professional mental health treatment or has been prescribed psych medications Dates of treatment? Was treatment completed? Is/was client adherent to treatment plan? Other comments:

RESEARCH ETHICS COMMITTEE
MBARARA UNIVERSITY OF SCIENCE AND TECHNOLOGY
SUCCEED WE MUST
P.O. BOX 1410, MBARARA
