# Supplementary material for: Adaptation of the Client Diagnostic Questionnaire for East Africa
Source: PLOS Glob Public Health. 2024 Mar 19;4(3):e0001756. doi: 10.1371/journal.pgph.0001756 (PMC10950255; doi:10.1371/journal.pgph.0001756)
Supplement: S1 Data — (DOCX) [file pgph.0001756.s004.docx]

Notes on East Africa IeDEA Analysis Datasets

**Project:** Adaptation of the Client Diagnostic Questionnaire for East Africa

**Concept Number:** 176b

**Principal Investigators:** Edith Kwobah

**Datasets Created:** ek_cdqvalidation

**Creation Date:** 19 Aug 2021

**Version:** 1.0

**Sites Included:** AMPATH, Mbarara, FACES

**Created By:** Steve Brown

**Statistician:** Constantin Yiannoutsos

**Cohort:** Syndemics participants completing the CDQ instrument who also were evaluated by a psychiatrist. N=90, 30 participants from each program.

**Notes:**

- *client_id* – Unique patient ID
- Psychiatrist review variables all contain “_psy” in the variable name, and “PSYCHIATRIST” in the label.
- *Program* identifies the site cohort for each participant
- All screening variables are Boolean, where 1=Yes/Present and 0=No/Absent

**Variables:**

| **Data Set Name** | ek_cdqvalidation | **Observations** | 90 |
| --- | --- | --- | --- |
| **Member Type** | DATA | **Variables** | 34 |
| **Engine** | V9 | **Indexes** | 0 |
| **Created** | 08/19/2021 10:36:00 | **Observation Length** | 248 |
| **Last Modified** | 08/19/2021 10:36:00 | **Deleted Observations** | 0 |
| **Protection** |  | **Compressed** | NO |
| **Data Set Type** |  | **Sorted** | NO |
| **Label** |  |  |  |
| **Data Representation** | WINDOWS_64 |  |  |
| **Encoding** | wlatin1 Western (Windows) |  |  |

| **Variables in Creation Order** | | | | | | |
| --- | --- | --- | --- | --- | --- | --- |
| **#** | **Variable** | **Type** | **Len** | **Format** | **Informat** | **Label** |
| **1** | client_id | Char | 7 | $7. | $7. |  |
| **2** | program | Char | 7 |  |  |  |
| **3** | client_no_positive_score_psy | Num | 8 | BEST12. | BEST32. | PSYCHIATRIST:No Positive Screen in any Module |
| **4** | depressive_disorder_psy1 | Num | 8 | BEST12. | BEST32. | PSYCHIATRIST: Positive for Major Depressive Syndrome |
| **5** | depressive_disorder_psy2 | Num | 8 | BEST12. | BEST32. | PSYCHIATRIST:Positive for Other Depressive Syndrome |
| **6** | anxiety_disorder_psy1 | Num | 8 | BEST12. | BEST32. | PSYCHIATRIST:Positive for Panic Syndrome |
| **7** | anxiety_disorder_psy2 | Num | 8 | BEST12. | BEST32. | PSYCHIATRIST:Positive for Generalized Anxiety Syndrome |
| **8** | alcohol_abuse_psy1 | Num | 8 | BEST12. | BEST32. | PSYCHIATRIST:Positive for Alcohol Abuse, past 6 months |
| **9** | alcohol_abuse_psy2 | Num | 8 | BEST12. | BEST32. | PSYCHIATRIST:Positive for Alcohol Abuse, past 30 days |
| **10** | drug_abuse_psy1 | Num | 8 | BEST12. | BEST32. | PSYCHIATRIST:Positive for Drug Abuse, past 6 months |
| **11** | drug_abuse_psy2 | Num | 8 | BEST12. | BEST32. | PSYCHIATRIST:Positive for Drug Abuse, past 30 days |
| **12** | post_traum_stress_psy1 | Num | 8 | BEST12. | BEST32. | PSYCHIATRIST:Positive on PTSD Screen |
| **13** | psychosis_psy1 | Num | 8 | BEST12. | BEST32. | PSYCHIATRIST:Positive on Psychosis Screen |
| **14** | treatment_experience_psy1 | Num | 8 | BEST12. | BEST32. | PSYCHIATRIST:Client has had professional mental health treatment or has been prescribed psych medications in the past 6 months |
| **15** | treatment_experience_psy2 | Num | 8 | BEST12. | BEST32. | PSYCHIATRIST:Client is currently receiving professional mental health treatment or has been prescribed psych medications |
| **16** | dd_tool_psy | Char | 4 |  |  | PSYCHIATRIST:Tool Utilized in diagnosis - Depression |
| **17** | ad_tool_psy | Char | 4 |  |  | PSYCHIATRIST:Tool Utilized in diagnosis - Anxiety |
| **18** | aa_tool_psy | Char | 4 |  |  | PSYCHIATRIST:Tool Utilized in diagnosis - Alcohol Abuse |
| **19** | da_tool_psy | Char | 4 |  |  | PSYCHIATRIST:Tool Utilized in diagnosis - Drug Abuse |
| **20** | ptsd_tool_psy | Char | 4 |  |  | PSYCHIATRIST:Tool Utilized in diagnosis - PTSD |
| **21** | ps_tool_psy | Char | 4 |  |  | PSYCHIATRIST:Tool Utilized in diagnosis - Psychosis |
| **22** | client_no_positive_score | Num | 8 | BEST12. | BEST32. | CDQ:No Positive Screen in any Module |
| **23** | depressive_disorder1 | Num | 8 | BEST12. | BEST32. | CDQ:Positive for Major Depressive Syndrome |
| **24** | depressive_disorder2 | Num | 8 | BEST12. | BEST32. | CDQ:Positive for Other Depressive Syndrome |
| **25** | anxiety_disorder1 | Num | 8 | BEST12. | BEST32. | CDQ:Positive for Panic Syndrome |
| **26** | anxiety_disorder2 | Num | 8 | BEST12. | BEST32. | CDQ:Positive for Generalized Anxiety Syndrome |
| **27** | alcohol_abuse1 | Num | 8 | BEST12. | BEST32. | CDQ:Positive for Alcohol Abuse, past 6 months |
| **28** | alcohol_abuse2 | Num | 8 | BEST12. | BEST32. | CDQ:Positive for Alcohol Abuse, past 30 days |
| **29** | drug_abuse1 | Num | 8 | BEST12. | BEST32. | CDQ:Positive for Drug Abuse, past 6 months |
| **30** | drug_abuse2 | Num | 8 | BEST12. | BEST32. | CDQ:Positive for Drug Abuse, past 30 days |
| **31** | post_traum_stress1 | Num | 8 | BEST12. | BEST32. | CDQ:Positive on PTSD Screen |
| **32** | psychosis1 | Num | 8 | BEST12. | BEST32. | CDQ:Positive on Psychosis Screen |
| **33** | treatment_experience1 | Num | 8 | BEST12. | BEST32. | CDQ:Client has had professional mental health treatment or has been prescribed psych medications in the past 6 months |
| **34** | treatment_experience2 | Num | 8 | BEST12. | BEST32. | CDQ:Client is currently receiving professional mental health treatment or has been prescribed psych medications |
