# Supplementary material for: Adaptation of the Client Diagnostic Questionnaire for East Africa
Source: PLOS Glob Public Health. 2024 Mar 19;4(3):e0001756. doi: 10.1371/journal.pgph.0001756 (PMC10950255; doi:10.1371/journal.pgph.0001756)
Supplement: Attachment — Submitted filename: Response to Reviewers.docx [file pgph.0001756.s006.docx]

**Response to Reviewers and journal requirements**

| **Journal requirements** | **Response** | **Line number** |
| --- | --- | --- |
| **Requirement 1.**  Our staff editors have determined that your manuscript is likely within the scope of our Global Mental Health: challenges, opportunities, and the future of the field. This editorial initiative is headed by a team of Guest Editors for PLOS GPH: Rochelle Burgess (University College of London) and Dixon Chibanda (University of Zimbabwe and London School of Tropical Medicine and Hygiene). The Collection invites researchers to submit original research which engages with, or disrupts, the urgent needs across the global mental health landscape. We especially encourage submissions of studies that critically interrogate the status quo of the field and that involve inter-/trans-disciplinary approaches and those which share perspectives from underrepresented global regions and communities. Additional information can be found on our announcement page: <https://collections.plos.org/call-for-papers/global-mental-health-opportunities-challenges/>  If you would like your manuscript to be considered for this collection, please let us know in your cover letter and we will ensure that your paper is treated as if you were responding to this call.  Please note that being considered for the Collection does not require additional peer review beyond the journal’s standard process and will not delay the publication of your manuscript if it is accepted by PLOS GPH. If you would prefer to remove your manuscript from collection consideration, please specify this in the cover letter." | **We would appreciate having our manuscript considered for this special edition.** | **N/A** |
| **Requirement 2.**  Please provide additional details regarding participant consent. In the ethics statement in the Methods and online submission information, please ensure that you have specified (1) whether consent was informed and (2) what type you obtained (for instance, written or verbal, and if verbal, how it was documented and witnessed). If your study included minors, state whether you obtained consent from parents or guardians. If the need for consent was waived by the ethics committee, please include this information. | **Patients ≥ 18 years who agreed to participate in the study were read a written consent form in their preferred language by a trained Research Assistant. Participants provided their name and signature on two forms, one for themselves and one for the study files. If a patient was unable to sign their name, a Witness (not affiliated with the study) was chosen to observe the consenting process. The participant then put their thumbprint in lieu of a signature and the Witness then signed and dated the consent.** | **187-191** |
| **Requirement 3**  Please amend your detailed online Financial Disclosure statement. This is published with the article. It must therefore be completed in full sentences and contain the exact wording you wish to be published.  a) State the initials, alongside each funding source, of each author to receive each grant. For example: "This work was supported by the National Institutes of Health (####### to AM; ###### to CJ) and the National Science Foundation (###### to AM)." c) State what role the funders took in the study. If the funders had no role in your study, please state: “The funders had no role in study design, data collection and analysis, decision to publish, or preparation of the manuscript.” | **We have amended our Financial Disclosure statement in the manuscript.** | **407-410** |
| **Requirement 4**  Please update your online Competing Interests statement. If you have no competing interests to declare, please state: “The authors have declared that no competing interests exist.” | **We have updated our Competing Interests statement in the manuscript.** | **406** |
| **Requirement 5**  In the online submission form, you indicated that "data used in this study is available on reasonable request". All PLOS journals now require all data underlying the findings described in their manuscript to be freely available to other researchers, either 1. In a public repository, 2. Within the manuscript itself, or 3. Uploaded as supplementary information. This policy applies to all data except where public deposition would breach compliance with the protocol approved by your research ethics board. If your data cannot be made publicly available for ethical or legal reasons (e.g., public availability would compromise patient privacy), please explain your reasons by return email and your exemption request will be escalated to the editor for approval. Your exemption request will be handled independently and will not hold up the peer review process, but will need to be resolved should your manuscript be accepted for publication. One of the Editorial team will then be in touch if there are any issues. | **We have provided our data set as part of our submission of Supplementary Information and have added it to the Legend.** | **516** |
| **Requirement 6**  We have noticed that you have uploaded Supporting Information files, but you have not included a list of legends. Please add a full list of legends for your Supporting Information files after the references list. | **We have added a Legend to the manuscript identifying all Supporting Information files.** | **504-516** |
| **Requirement 7**  Please review your reference list to ensure that it is complete and correct. If you have cited papers that have been retracted, please include the rationale for doing so in the manuscript text, or remove these references and replace them with relevant current references. Any changes to the reference list should be mentioned in the rebuttal letter that accompanies your revised manuscript. If you need to cite a retracted article, indicate the article’s retracted status in the References list and also include a citation and full reference for the retraction notice. | **We have reviewed our reference list and are unaware that any referenced manuscripts have been retracted. We did delete a repeated reference by Aidala et.al.** | **N/A** |
| **Reviewer 1 Comments** | **Response** | **Line number** |
| **Comment 1** Please provide a justification for the sample size you chose of 90 participants. | **We have included a justification for our sample size for translation and criterion validation of a questionnaire based on other studies doing cultural adaptations of health-related questionnaires (Tsang 2017); . We believe that a sample size of 90 participants is appropriate for this type of work as does not attempt to validate the content of the questionnaire itself.** | **183-187** |
| **Comment 2** Table 1 is very confusing. The column labeling is not clear and uniform. Why is there an empty column between EMPATH and FACES and what do these column labels even mean? | **We have revised the table name and column labeling for clarity. We have deleted the erroneous empty column.** | **173-175** |
| **Comment 3** Line 164-165; was additional consent sought for this particular study and not only the main study? State this clearly. | **Consent to participate in the validation activities was included in the main study (Syndemics) consent. Description of this consent has been expanded within the manuscript.** | **187-191** |
| **Comment 4** It is not clear which CDQ you are referring to at the different stages it was administered i.e. (the locally adapted one or the original CDQ). For example, in the criterion validation, the first administration by RA is assumed to be the final version of the CDQ translated to the local language (presumably), and when the physiatrist sees the same patient and administers the DSMV, and fills the CDQ summary sheet. It is not apparent whether it is the translated one or the original one in English. Please clarify. | **We have identified whether the CDQ referenced is the “original” or “locally translated” version in order to provide clarity throughout the manuscript. We have also clarified the documents used by the psychiatrists to evaluate the participants and record their diagnoses.** | **69**  **75**  **104**  **114**  **151**  **157**  **160**  **162**  **171-173**  **182**  **192**  **225-227**  **230**  **240**  **285-286**  **325**  **328**  **511** |
| **Comment 5** In assessing the validity of the translated CDQ we compare it with diagnosis by a specialist who is the gold standard in this case. Can you further explore how much the difference in the cadre of the interviewer affects this outcome? | **We have explored how the difference in diagnoses may be affected by inter-rater variability and its subsequent effect on the accuracy of between psychiatrists and the research assistants.** | **361-375** |
| **Comment 6** Line 196, which 11 items?? You did not mention them before | **This was to reference the 12 areas scored on the CDQ. They have been listed in the section on Criterion Validation.** | **193-199** |
| **Comment 7** Line 244, is it 3 or 4 languages?? You spoke of 4 before, why mention 3 | **Runyankole-Rukiga is a single language shared by two neighboring tribes in southwestern Uganda. Along with Swahili and Duhluo the total number of languages is three. We have clarified this in the Setting.** | **140**  **145**  **147**  **285** |
| **Comment 8** In your discussion, you have not compared the sensitivity, specificity and overall accuracy of the locally adapted CDQ to that of the original English version and explore what it means. Comparison was with a South African study which would do good to come after this. | **We have added the comparison of the local CDQ results to those of the original English version and have explored the differences between the two and their implications.** | **324-325** |
| **Reviewer 2 comments** | **Response** | **Line number** |
| **Comment 1**  There is not enough interrogation of the process of forward and back translation of tools developed for the needs of clinicians and patients in non-Western countries. Linguistic factors are important and the authors do consider these carefully. However, culture is more than simply language and language itself is used fluidly. It would be better to situate the paper in a cultural context as well as a linguistic one. I recommend reading the following open source paper Weekes BSH. Aphasia in Alzheimer’s Disease and Other Dementias (ADOD): Evidence From Chinese. American Journal of Alzheimer’s Disease & Other Dementias®. 2020;35. doi:10.1177/1533317520949708 | **We have added more language with the Methods and Discussion to better address both semantic and conceptual equivalence and to emphasize the importance of cultural input into the translations of a questionnaire.** | **156-168**  **331-336** |
| **Comment 2**  There are minor errors of punctuation and spelling. I would be happy to proof read another copy to help. | **We have carefully reviewed the manuscript and have made corrections to spelling, punctuation errors. We have made some minor edits in wording for clarification.** | **All lines.** |
